# Beyond spikes: Multiscale computational analysis of *in vivo* long-term recordings in the cockroach circadian clock

**DOI:** 10.1162/netn_a_00106

**Published:** 2019-09-01

**Authors:** Pablo Rojas, Jenny A. Plath, Julia Gestrich, Bharath Ananthasubramaniam, Martin E. Garcia, Hanspeter Herzel, Monika Stengl

**Affiliations:** Theoretical Physics, University of Kassel, Kassel, Germany; Animal Physiology, University of Kassel, Kassel, Germany; Animal Physiology, University of Kassel, Kassel, Germany; Institute for Theoretical Biology, Humboldt University of Berlin and Charité Universitätsmedizin, Berlin, Germany; Theoretical Physics, University of Kassel, Kassel, Germany; Center for Interdisciplinary Nanostructure Science and Technology (CINSaT), University of Kassel, Kassel, Germany; Institute for Theoretical Biology, Humboldt University of Berlin and Charité Universitätsmedizin, Berlin, Germany; Animal Physiology, University of Kassel, Kassel, Germany; Center for Interdisciplinary Nanostructure Science and Technology (CINSaT), University of Kassel, Kassel, Germany

**Keywords:** Circadian and ultradian rhythms, Neuropeptides, Event detection, Wavelet

## Abstract

The circadian clock of the nocturnal Madeira cockroach is located in the accessory medulla, a small nonretinotopic neuropil in the brain’s visual system. The clock comprises about 240 neurons that control rhythms in physiology and behavior such as sleep-wake cycles. The clock neurons contain an abundant number of partly colocalized neuropeptides, among them pigment-dispersing factor (PDF), the insects’ most important circadian coupling signal that controls sleep-wake rhythms. We performed long-term loose-patch clamp recordings under 12:12-hr light-dark cycles in the cockroach clock *in vivo*. A wide range of timescales, from milliseconds to seconds, were found in spike and field potential patterns. We developed a framework of wavelet transform–based methods to detect these multiscale electrical events. We analyzed frequencies and patterns of events with interesting dynamic features, such as mixed-mode oscillations reminiscent of sharp-wave ripples. Oscillations in the beta/gamma frequency range (20–40 Hz) were observed to rise at dawn, when PDF is released, peaking just before the onset of locomotor activity of the nocturnal cockroach. We expect that *in vivo* electrophysiological recordings combined with neuropeptide/antagonist applications and behavioral analysis will determine whether specific patterns of electrical activity recorded in the network of the cockroach circadian clock are causally related to neuropeptide-dependent control of behavior.

## Author Summary

We seek to understand how the neuronal network of the cockroach circadian clock controls temporal rhythms in physiology and behavior. Circadian clocks in mammals and insects alike employ neuropeptides for information processing that bypass synaptic information transfer. Neuropeptides appear to work as coupling factors synchronizing and binding neuronal circuits on multiple timescales, employing schemes of temporal encoding. Expanding known techniques with novel approaches, we describe oscillations of electrical events on multiple timescales in long-term *in vivo* electrophysiological recordings. In future studies, we will combine experimental approaches with the construction of minimal oscillator networks to examine how the cockroach brain controls sleep-wake cycles based on neuropeptide signaling.

## INTRODUCTION

**Figure 1.**
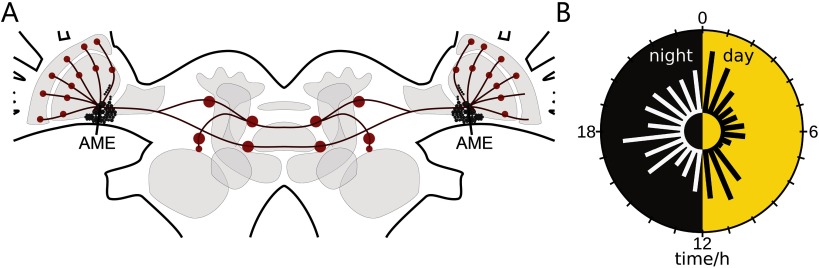
The cockroach circadian clock with innervating pigment-dispersing factor (PDF)-expressing clock neurons control circadian sleep-wake rhythms in synchrony with the light-dark cycle. (A) Schematic of the circadian network (red dots; neuropils) of PDF circadian clock neurons in the cockroach brain. The circadian clock of the nocturnal Madeira cockroach is the accessory medulla (AME) ventromedial to the medulla in the brain’s optic lobes. It is innervated by about 240 adjacent neuropeptidergic clock neurons (small black filled circles). Among them are 12 PDF neurons. They are clock in- and outputs; they couple the bilaterally symmetric clocks, controlling sleep-wake cycles (modified after Stengl et al., 2015, reprinted with permission from Elsevier). (B) Extracellular recordings of the isolated AME in vitro revealed that endogenously generated electrical activity changes systematically during the course of the day with peaks at dusk and dawn, as well as during the middle of night and day. (modified after Stengl & Arendt, 2016, reprinted with permission from Elsevier).

Rhythms in animals are generated by complex interplays of molecular and cellular feedback loops (biological clocks) and also by network synchronizations producing temporally structured outputs. Biological circadian clocks control rhythms in physiology and behavior synchronized to the 24-hr light-dark cycle of the environment. The Madeira cockroach (*Rhyparobia maderae*) is an established model system for chronobiology (Nishiitsutsuji-Uwo & Pittendrigh, 1968; Page, 1984; Stengl, Werckenthin, & Wei, 2015). Madeira cockroaches are relatively long-lived with a life span of up to 2.5 years. They are nocturnal animals, since they restrict their activity to the dark night, while they rest (sleep) during the light phase of each day. Its circadian clock is the accessory medulla (AME), a small glomerular neuropil ventromedial to the medulla in the brain’s optic lobes (Figure 1A; Reischig & Stengl, 2003a; Stengl & Homberg, 1994). The AME is innervated by about 240 neuropeptidergic clock neurons of mostly unknown function (Reischig & Stengl, 2003b). Best studied are the pigment-dispersing factor (PDF)-expressing clock neurons that control circadian sleep-wake rhythms not only in the cockroach, but also in other insects such as the fruit fly *Drosophila melanogaster* (Figure 1A; reviews: Hermann-Luibl & Helfrich-Foerster, 2015; Stengl & Arendt, 2016). The actions of PDF in the insect circadian clock (reviews: Stengl & Arendt, 2016; Stengl et al., 2015) reflect actions of vasoactive intestinal polypeptide (VIP) in the mammalian circadian clock (Patton & Hastings, 2018; Vosko, Schroeder, Loh, & Colwell, 2007). Next to resemblance of PDF’s and VIP’s circadian functions, the cellular and molecular organization of the cockroach and the mammalian clock also resemble each other (Vansteensel, Michel, & Meijer, 2008). Intriguingly, both clocks are abundant with neuropeptides that do not require direct synaptic connectivity (Patton & Hastings, 2018). Instead, neuropeptides perform volume transmission. Neuropeptides are stored in dense core vesicles in the cells and are usually not released only at synaptic sites into the synaptic cleft. They are released at multiple sites of the neuron, into the respective carrier medium. In the volume of the extracellular space, the neuropeptides can be carried very far, acting over extended time spans, depending on their life time. Thus, neuropeptides bind spatially distributed neuropeptide-receptor-expressing neurons into an ensemble with common, synchronous activity. Since neuropeptide-dependent ensembles of neurons spike synchronously, they generate distinct electrical patterns that can be detected in extracellular recordings (Schneider & Stengl, 2005). Also, on the single cell level neuropeptidergic neurons can produce characteristic electrical signatures. Neuropeptidergic neurons in mammals and insects were shown to express ultradian membrane potential oscillations, generating bursts of action potentials during release of their respective neuropeptides (Hatton, 1982; Kamimoto, Nohara, & Ichikawa, 2006; Wei et al., 2014). Despite the fact that neuropeptides express these characteristics and despite their abundant quantity in brains of evolutionary widely diverse species, their mechanisms of actions are not well understood. Therefore, circadian clocks with their numerous colocalized neuropeptides are well suited for the study of neuropeptide actions/functions in general.

We aspire to understand why neuropeptidergic clock neurons express ultradian and circadian rhythmicity and how rhythmic activity on multiple timescales is orchestrated to enable circadian clock functions. The cockroach clock contains about 100 times fewer, larger neurons than the mammalian clock. Thus, it is more easily accessible to electrophysiological and neurochemical analysis, even at the level of single identified cells (Loesel & Homberg, 2001). Already a complete 3-D atlas of the cockroach brain was reconstructed with arborizations of PDF-clock neurons embedded (Wei, el Jundi, Homberg, & Stengl, 2010). Based upon these advantages we work with the cockroach for the electrophysiological analysis of neuropeptide actions on the circadian clocks’ cellular level.

Here, extracellular long-term loose-patch clamp recordings from the AME were performed in the intact animal over more than 24 hr. Thus, for the first time, we gained information about recurring events, oscillations, and network dynamics from a circadian clock receiving sensory information from the compound eyes, as well as phase information from both bilaterally symmetric clocks in the cockroach. Based upon these *in vivo* recordings, we developed a framework for the analysis of the activity of the circadian clock network over different timescales. Scales ranged from action potential firing of a few milliseconds, to local field potentials, and dynamic features of sequences of events ranging from seconds, to minutes, to hours. The focus was not the analysis of action potential patterns of single neurons. Rather, we searched for Zeitgeber time (ZT)-specific changes in activity patterns indicative of rhythmic release of neuropeptides (Figure 1B). Evidence is accumulating that during the day the clock releases the neuropeptide PDF with endogenous rhythmicity. In turn, PDF generates and controls antagonistic neuronal ensembles: the sleep and the arousal circuits, which are phase-coupled to the external light-dark cycle (Chatterjee et al., 2018; Gestrich et al., 2018; Schneider & Stengl, 2005; Wei & Stengl, 2011). We want to know whether the hypothesis of clock-controlled neuropeptide-dependent ensemble formation (Schneider & Stengl, 2005; Stengl & Arendt, 2016; Stengl et al., 2015) finds support *in vivo* in our noninvasive long-term recordings of the cockroach clock. Therefore, we searched for signatures of synchronized electrical activity during the light phase consistent with light- and clock-controlled PDF-dependent ensemble formation.

## RESULTS

**Figure 2.**
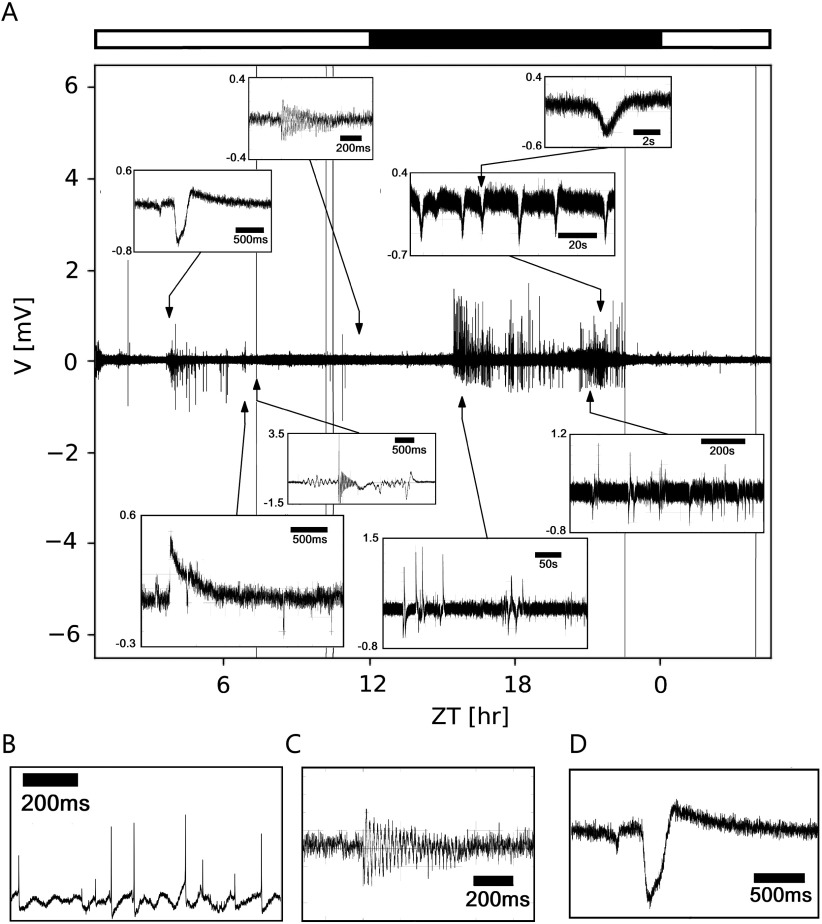
Multiscale events found in an *in vivo* long-term loose-patch clamp recording from the cockroach circadian clock, the accessory medulla. (A) Duration of events ranged from a few milliseconds (ms) to seconds (s) and minutes (min), partially overlapping each other. According to their duration and pattern three different classes of events were distinguished: first: very fast events of a few ms (B, action potentials); second: events that can be approximated by sustained sinusoidal oscillations occurring over hundreds of ms up to several s, or min; (C); third: events that are of limited duration, between the previous two extreme cases that sometimes exhibit oscillatory behavior (D).

To search for Zeitgeber time (ZT)-dependent changes of activity in a circadian clock that are indicative of neuropeptide actions, we performed 24- to 48-hr-long *in vivo* loose-patch clamp (∼ 1 Gigaohm seal) recordings of the accessory medulla (AME), the circadian clock of the Madeira cockroach (*n* = 18). These electrophysiological recordings contained a wide range of events at multiple time-scales from single action potentials of 1−2 ms to second-long field potentials. Furthermore, events were found to form episodic patterns in the range of seconds to hours (Figure 2A).

Electrophysiological recordings can be regarded as time series, composed of a sequence of events embedded in a noisy signal, which may also contain oscillations. Event detection methods were developed in different fields (Guralnik & Srivastava, 1999; Lilly, 2017; Merel, Shababo, Naka, Adesnik, & Paninski, 2016; Tu, Hwang, & Ho, 2005). Usually, they treated an event as one of three classes of objects, depending on the support in the time and spectral domains (duration, frequency): (a) events that are singularities, that is, events of very short duration (Figure 2B), such as action potentials, (b) events that can be approximated by sustained sinusoidal oscillations and that are elongated in the time axis (Figure 2C), such as autoreceptor-dependent neuropeptide release; or (c) events that are between those two extreme cases, localized in time, showing a limited duration, and sometimes exhibiting oscillatory behavior (Figure 2D), as synaptic events (Guzman, Schlögl, & Schmidt-Hieber, 2014; Leise, 2013; Lilly & Olhede, 2009; Masimore, Kakalios, & Redish, 2004; Pernía-Andrade et al., 2012; Principe & Brockmeier, 2015; Rey, Pedreira, & Quiroga, 2015; Richardson & Silberberg, 2008; Shi, Nenadic, & Xu, 2010; Tu et al., 2005). The detection of individual events and the measurement of their specific characteristics, such as amplitude, duration, and waveform features, are of interest in the study of electrophysiological signals that contain both synaptic events and spikes (Guzman et al., 2014; Merel et al., 2016; Pernía-Andrade et al., 2012; Richardson & Silberberg, 2008; Shi et al., 2010). One of the main limitations of current detection methods is that they focus on the analysis of either action potentials or synaptic events. Thus, the durations of events are assumed to be in a relatively narrow range, usually within the same order of magnitude. Here, we relaxed this condition to events with durations over at least three orders of magnitude.

### Reliable Detection of Multiscale Events With Wavelet Transform–Based Methods

To quantify and analyze these complex multiscale data, we used two different approaches. For segments that presented a narrow range of event durations, we performed methods based on conventional spike train analysis. For analysis of recordings containing a wider range of event durations, we developed a multiscale approach based on the wavelet transform (Supporting Information, Figures S2–S7). This approach allowed us to detect events at multiple timescales of about three orders of magnitude and to extract their basic features, such as duration and amplitude. Next, we examined whether event patterns and oscillations at different ultradian timescales also expressed circadian timescales, occurring at consecutive days at the same ZT.

**Figure 3.**
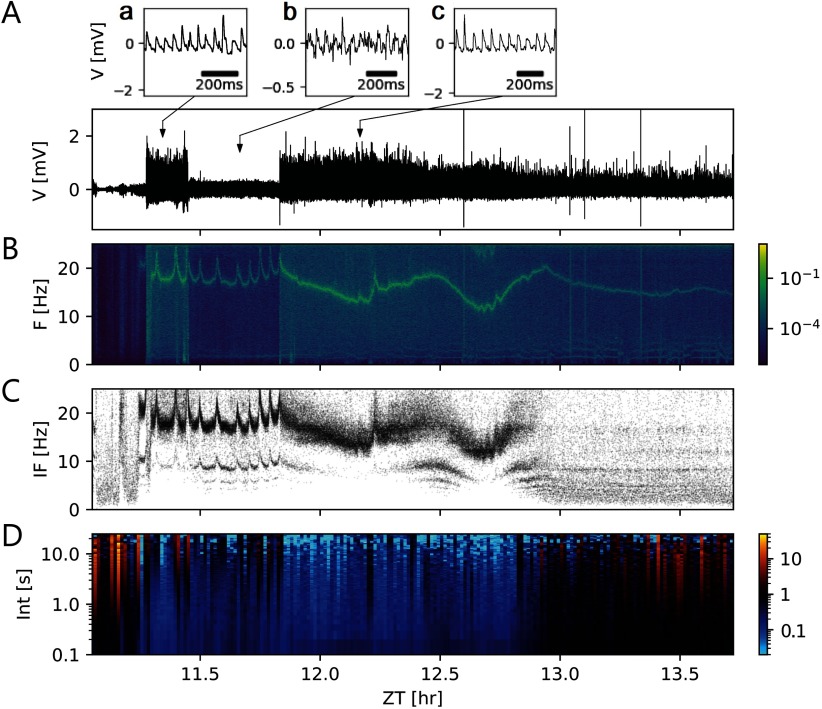
Scale-blind analysis of events requires much manual tuning of parameters and might not be reliable for this kind of multiscale data. (A–D) Scale-blind analysis of a segment from a loose-patch clamp recording with nonoverlapping events of comparable scales. These events can be detected by applying a threshold and finding the peaks that surpass it. Thus, a single event train is obtained, suitable for conventional spike train analysis. (A) Segment of 2.6-hr loose-patch clamp recording between Zeitgeber time (ZT) 11.1 and ZT 13.7. Following Equation 3, threshold parameters required laborious manual tuning between 1 and 3, as compared with the usual 3–5 range, because the duration of the events are comparable to their interevent intervals, as insets A–C show. (B) Spectrogram of the *in vivo* recording. (C) Instantaneous firing frequency of the recording, as the inverse of the interevent interval. A tonic stable firing frequency between 12 and 20 Hz is recognizable as a band of points. However, missing events (false negatives) and spurious events (false positives) produce less concentrated bands and a second band. Errors in event detection may be due to a small signal-to-noise ratio or an incorrect threshold estimation. The firing frequency of the main band correlates to the main frequency in the spectrogram. This also indicates that the durations of the events are comparable to the interevent interval. This violates the assumption of well-separated events. (D) Time-resolved Fano factor of the event train as a metric for event train variability. Smaller values are an indication of a tonic regular firing, while larger values indicate irregular firing. A Fano factor of 1 indicates an event train variability equivalent to a Poisson process. The x-axis shows ZTs, while the y-axis indicates the time windows taken for the Fano factor calculation.

Threshold detection was applied for a recording (Figure 3A) that presented a narrow range of timescales of events. The threshold parameter (Equation 3) was manually set to values between 1 and 3, where the recording showed stationarity. Compared with the usual choices in the range 3–5, the values for the threshold parameter were smaller because the duration of events were comparable to their interevent intervals. Despite the threshold calculation being dynamic and adapted to properties of noise, it was not possible to set a unique threshold parameter that worked for the whole recording. Therefore, the above-mentioned manual tuning of the method was very time-consuming.

We performed spike train analysis on the event trains obtained. For instance, spectrogram analysis (Figure 3B) and instantaneous frequency plots (Figure 3C) were created. The same firing frequency at each point was clearly distinguishable, as a line of greater power density in the spectrogram of the signal (Figure 3B). Dense bands in the instantaneous frequency plot indicated a stable firing frequency over consecutive events and pointed towards ensembles of neurons firing with the same frequency at the same or integer multiples of the same frequency and the same phase (Figure 3C). This is another indication that the event durations were comparable to the respective interevent intervals and that the entire signal was strongly periodic. As a consequence, threshold parameters had to be applied outside their usual range: As events become wide enough, the assumption of the events being well separated and punctual was no longer valid. Time-resolved Fano factor of the event trains supplied a metric for event train variability (Figure 3D). In this case, both tonic regular firing as well as irregular firing, were present in the recording.

**Figure 4.**
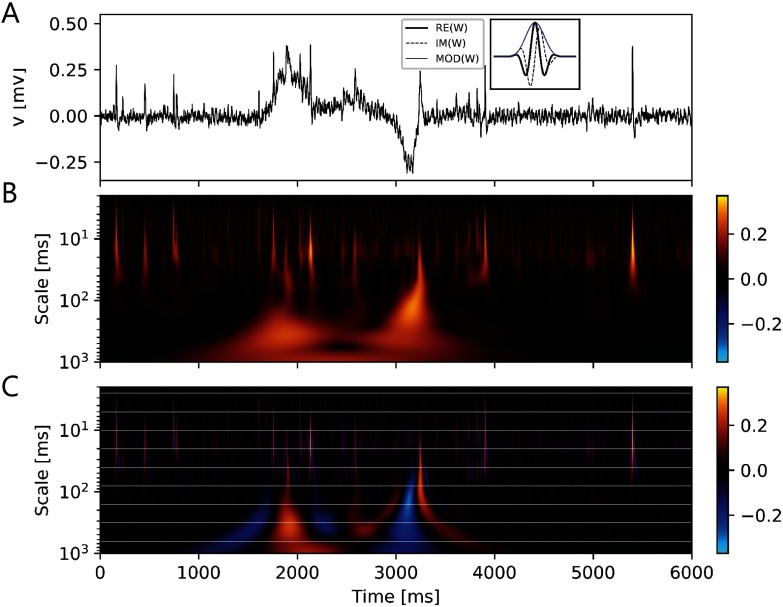
Wavelet transform allows clear detection and separation of electrical events of different durations such as fast action potentials (∼2 ms) and slower field potentials (∼50 ms to several seconds). Furthermore, it estimates polarity and amplitudes of events. Example of event detection in the *in vivo* loose-patch clamp recording using wavelet transform (A). Morse wavelet (inset) was used to detect time-localized events in segments of 6 s. Analytic wavelet transform (AWT) of the signal showed local maxima and minima at the time of events. (A) Six sec of neuronal activity at ZT 16.7. Inset: Modulus, real and imaginary part of an analytic Morse wavelet, using *β* = 2 and *γ* = 3. *β* and *γ* control the shape and spectral properties of the wavelet, especially its limits in the time and frequency domains. With higher values of *β* and *γ*, frequency components decay more sharply from its peak frequency, leading to a narrower bandwidth. (B) Modulus of the AWT. Events could be detected by searching for local maxima and minima in the modulus of the AWT, but the method lost resolution in detecting individual continuous events of opposite polarity. Amplitudes of events were indicated in the color scale. (C) Real part of the AWT. Individual continuous events could be separately recognized. Polarity and amplitude of events were indicated in the color scale. Event detections could also be performed using the subset of scale values shown in horizontal white lines. By selecting only scales from a subset of *s* = 2^*j*^Δ*T*, *j* = 0, 1, …, *N*_*s*_, computational cost was reduced, at the expense of less scale resolution. Thus, each of the scales in the subset became representative of a bandwidth of scales. This procedure enabled affordable processing times for large datasets. For peaks in the wavelet transform that were relevant in more than one consecutive scale, the winner was selected based on absolute values.

As the conventional methods for spike train analysis did not reliably separate events, a wavelet transform–based method for event detection was applied to the long-term *in vivo* recordings. Events were detected by locating the maxima and minima of the modulus of the analytical wavelet transform (AWT, Figures 4A–C). The method performed well in detecting overlapping events of different durations and amplitudes. Furthermore, it provided estimates for these features and also recognized continuous events when the real part of the AWT was used instead. As input parameters, only the maximum and minimum durations were required. Thus, all the scales between these extremes were automatically defined following a geometric sequence.

### Screening of Activity Intensity Heatmaps Revealed Events, Episodes, and Network Dynamics from Milliseconds to Hours

**Figure 5.**
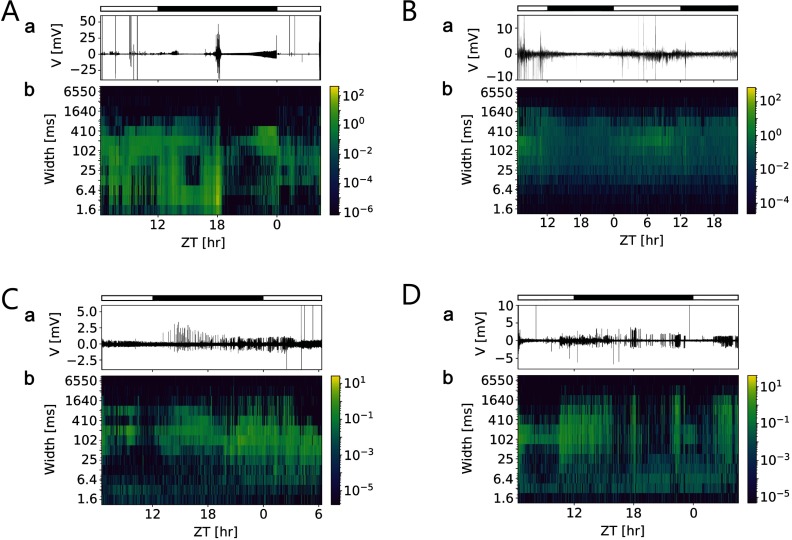
Multiscale activity heatmaps of four different long-term *in vivo* loose-patch clamp recordings of the cockroach clock reveal daytime-dependent changes of field potentials that were not driven by the light-dark cycle. (A–D, a) Traces of original loose-patch clamp recordings in a 12:12-hr light (open bar) – dark (filled bar) cycle. (A–D, b) Coarse grain activity heatmaps of events at different durations (widths, ms) in the recordings. For each bin of 100 s, events of the same duration were counted and weighted by their amplitudes (color coded). This enabled to determination of whether there are Zeitgeber time (ZT)-dependent events of specific durations that occur with circadian periods, either directly driven by the light-dark cycle, or via endogenous rhythms. This serves as an exploratory analysis to find events of potential biological significance such as field potential changes at dusk when the animal starts to become active. Only in (A) some of the events coincided with lights on or lights off. Otherwise, either specific electrical events preceded the day (B, C) or night phase (A, D), indicative of its endogenous rhythmicity.

**Figure 6.**
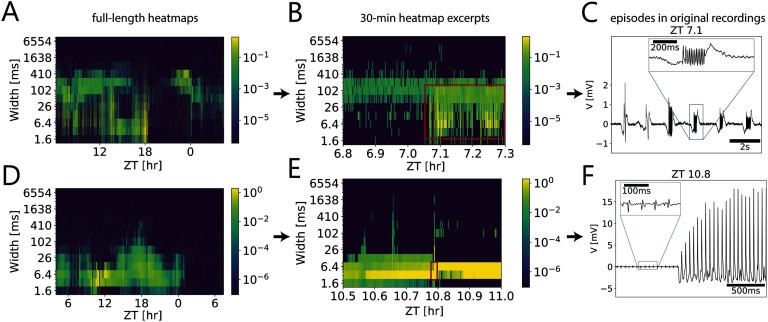
Multiscale activity heatmaps as a tool for detecting interesting episodes in the underlying dynamical system. (A, D) Full-length coarse grain activity heatmaps as well as 30-min time windows (B, E) that were further up-scaled (C, F) to reveal electrical events at different durations (widths) in the *in vivo* long-term patch clamp recordings of the cockroach circadian clock. For each bin of 100 s, the number of events of the same duration were counted and weighted by their amplitudes (color coded). (A–C) Larger and longer episodic events coincided with/were followed by oscillations of increasing amplitude that could be identified by different widths (red rectangle in B). (D–F) The onset of positive excursions resembled a Hopf bifurcation in the parameter space of the underlying dynamical system (red renctangle in E; Baer, Erneux, & Rinzel, 1989. Firing frequency was constant during this process.

**Figure 7.**
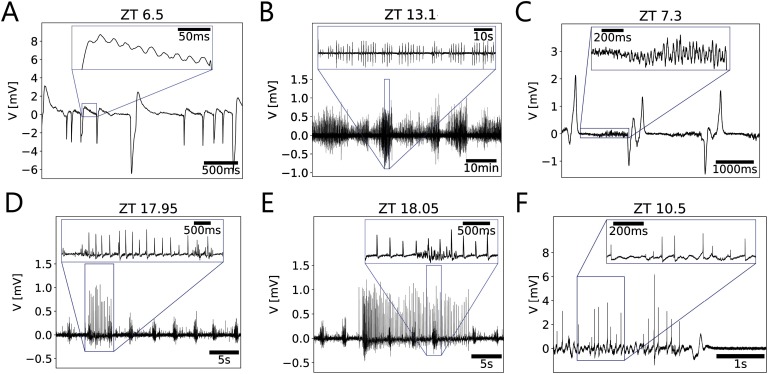
Exploratory analysis of multiscale activity heatmaps revealed episodes in the recordings, as indications of synchronizations and bifurcations in the underlying dynamical system. (A) Oscillations appeared to be followed by larger amplitude events. (B) Amplitude modulation was present at two timescales (10 s and 10 min), resembling a beating-like behavior of the envelopes of the oscillations. (C) Oscillations of increasing amplitudes preceded larger and broader episodic events. (D, E) Episodic bursts of small-amplitude events occurred phase-locked to initiation and termination of larger amplitude tonic firing. (F) Small spikes in the range of action potentials added up to larger events apparently due to synchronization of different neuronal units.

After identifying events using wavelet transform–based methods, we used an exploratory approach of visualizing activity at multiple timescales in activity intensity heatmaps (Figures 5A–D; Figures 6A, D). In these maps, activity intensity is calculated for each scale as the sum of the amplitudes of the events over a time window of 100 s. This allowed us to visually compare the individual activity patterns and to find characteristic events at specific ZTs. Activity patterns identified in the full-length heatmaps (Figures 6A, D) were magnified further to inspect dynamics of the activity pattern in 30-min heatmap excerpts (Figures 6B, E). In a next step, various events with interesting patterns were isolated from the original recording trace, which could be indicators of specific structural configurations such as bifurcations (Baer et al., 1989) in the underlying dynamical system (Figures 6C, F; Figures 7A–F). For example, oscillations were observed to precede the onset of large-amplitude events (Figure 6C; Figure 7C). This pattern is comparable to intermittency (Stavrinides & Anagnostopoulos, 2013; Żebrowski & Baranowski, 2004). Onsets of sustained oscillations resembling Hopf bifurcations were identified (Figure 6F). Mixed-mode oscillations reminiscent of sharp-wave ripples were found to coexist with larger amplitude events (Figure 7A). Furthermore, amplitude modulations of events were identified to occur concurrently at different timescales, in the range of 10 s as well as in the range of 10 min (Figure 7B). Also, apparently phase coupling of two different neuronal units was detected, one firing regularly and the other firing intermittently with low-intensity bursts (Figures 7D, E). Interestingly, the firing of the tonic unit seemed to be phase-coupled to the bursts from the other unit. These observations served to hint at the structural configuration of the system and will be helpful for further analysis of connectivity of the cockroach circadian clock’s neural network. To infer the possible interrelations of events, the events were grouped additionally by similarities through a clustering process (Lara, Lizcano, Pérez, & Valente, 2014) (Figure S1). Bayesian Gaussian mixtures were applied, using the estimated duration, amplitude, and time localization as coordinates. As a result, events were clustered and labeled in groups that were coherent with visual inspection of raw data.

### Ultradian Oscillations in the Alpha, Beta, and Gamma Range Also Expressed Circadian Rhythmicity in the Cockroach Clock

**Figure 8.**
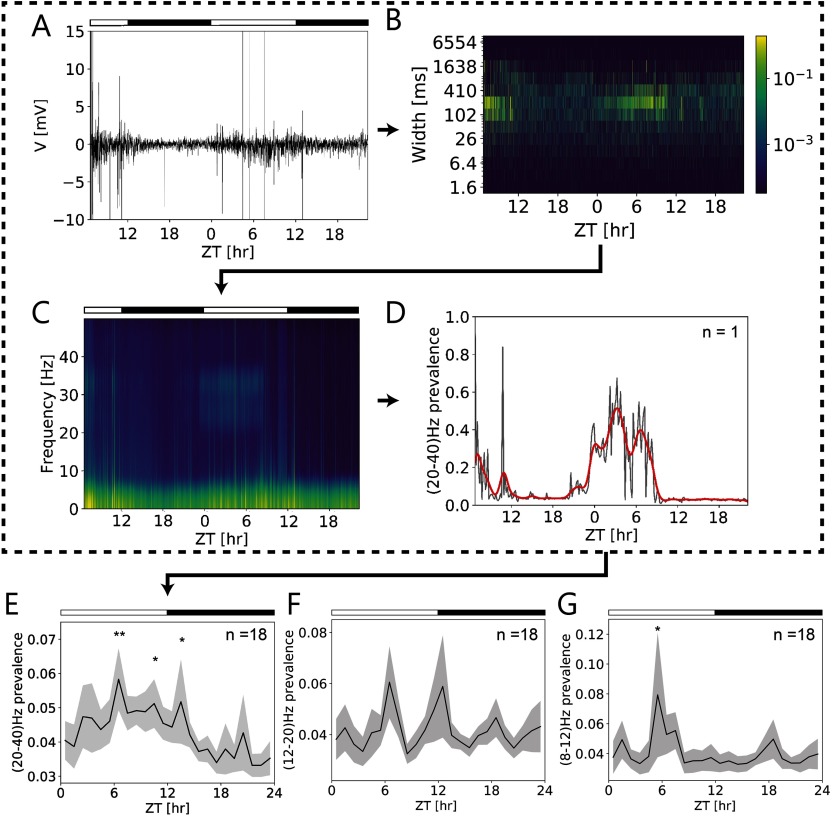
Multiscale frequencies at ultradian (ms) and circadian (∼24 hr) timescales present in long-term *in vivo* loose-patch clamp recordings of the cockroach circadian clock. (A) Original *in vivo* loose-patch clamp recording over the course of almost 2 days in 12:12 hr light (open bar)–dark (filled bar) cycle. (B) Full-length heatmap (of A) indicated strong activity increases of events of several hundred ms durations at ∼ZT 7 at both days. (C) Spectrogram of the same recording revealed multiple rhythms at different Zeitgeber times (ZTs), including ZT-dependent oscillations in the beta/gamma frequency range (∼ 20–40-Hz). (D) The 20- to 40-Hz oscillations increased in prevalence already before lights on (at dusk) with multiple peaks (∼ZTs 6.50, 10.5 (day 1), 0.5, 3, and 6.5 (day 2)) during the day, about 2 hrs apart. It declined to zero prevalence preceding lights off (at ∼ZT 9). (E) ZT-dependent prevalence of beta/gamma oscillations expressed significant rhythmicity on the circadian timescale in all long-term *in vivo* recordings (*n* = 18). Significant peaks occurred during the day at ZTs 6.5 and 10.5, and at dusk at ZT 13.5 (*p* < 0.05; Table S1) (*n* = 18). (F) Also, prevalence of beta oscillations (12- to 20-Hz range) changed over the course of the day with a significant 6-hr periodicity (*n* = 18) (*p* = 0.036). (G) Furthermore, prevalence of alpha oscillations (8- to 12-Hz range) expressed significant rhythmicity on the circadian timescale with a peak at ZT 5.5 (*n* = 18) (*p* < 0.05; Table S1).

We searched for alpha (8–12 Hz), beta (12–28 Hz), and gamma (> 30 Hz) band oscillations in the cockroach clock that were observed before in electroencephalograms (EEGs) of the mammalian brain (Buzsáki & Draguhn, 2004; Khanna, Pascual-Leone, Michel, & Farzan, 2015). We wanted to determine whether oscillations in different frequency bands are a general property of neural networks with specific functional connectivity already present in the cockroach brain. Dominating frequency bands and their prevalence at certain ZTs were extracted from overall activity patterns of the AME recorded *in vivo* (Figures 8A–C). A heatmap generated from a single 2-day-long *in vivo* recording (Figures 8A, B) illustrated a strong increase in activity around midday. The spectrogram of this recording revealed a prevalence of power in a frequency band in the beta/gamma range (20–40 Hz; Figure 8C) during the light phase. The rise of its prevalence preceded lights on at ZT 0 and, thus, was not driven by light. It sharply declined at ZT 9, thus, it was not correlated with lights off at ZT 12 (Figure 8D). Several peaks of different amplitudes at apparently regular intervals were observed at ZT 6.5 and ZT 10.5 the first day and ZT 0.5, ZT 3, and ZT 6.5 the second day. When the prevalence of 20–40-Hz oscillations was examined in all long-term *in vivo* recordings (*n* = 18, Figure 8E), they were found to express rhythmicity also at circadian timescale. Significant maxima were observed at the middle of the day and at dusk (ZT 6.5, ZT 10.5, ZT 13.5, linear mixed model, *p* < 0.05, Table S1), with a steep decline at the middle of the night (Figure 8E). Prevalence of the beta/gamma frequency was significantly higher during the day compared with the night (linear mixed model, *p* < 0.05, Table S1). Additionally, we found ultradian 6-hr periodicity at 12–20 Hz (beta frequency band) prevalence (Figure 8F; ANOVA and sine fits, *p* = 0.036). In the alpha band range (8–12 Hz) the prevalence peaked significantly at ZT 5.5 (linear mixed model, *p* < 0.05, Table S1) at the middle of the day (Figure 8G). Thus, ultradian rhythms in different frequency bands observed before in mammalian brains were present in the cockroach brain and were gated by the circadian clock of the cockroach.

## DISCUSSION

Seeking to understand neuropeptide-dependent network characteristics of circadian clocks on multiple timescales, we employed long-term loose-patch clamp recordings *in vivo* of the circadian clock of the Madeira cockroach. Clock neurons of cockroaches and mammals alike contain an astounding abundance of neuropeptides (reviews: Patton & Hastings, 2018, Stengl & Arendt, 2016, Vosko et al., 2007). Thus, Zeitgeber time (ZT)-dependent neuropeptide release appears to be instrumental for circadian clock functions. Consequently, rather than studying action potential activity of single clock cells we focused on the analysis of electrical events that could be key signatures of neuropeptide functions, such as ensembles firing regularly and synchronously at ultradian frequency bands. With novel approaches based on the wavelet transform and activity heatmaps, we were able to detect and disaggregate events and event patterns over multiple timescales. Thereby, we revealed ultradian periodicities occurring at specific ZTs in the cockroach clock. We found ultradian rhythms in the alpha, beta, and gamma frequency ranges that also showed rhythmicity on the 24-h circadian timescale and intermediate timescales. In the majority of the *in vivo* clock recordings, 20- to 40-Hz rhythms were most common during the middle of the day and at dusk. Thus, they occurred at ZTs when endogenously rhythmic release of PDF was suggested to take place, phase-controlled via dusk and dawn (reviews: Stengl & Arendt, 2016; Stengl et al., 2015). Future analysis of long-term *in vivo* recordings combined with pharmacology will test whether gamma band rhythms are signatures of PDF actions in the cockroach clock controlling sleep during the day and arousal at dusk. Concurrently, we model a potential circadian clock network that comprises features found *in vivo* to allow for quantitative predictions of clock network characteristics and neuropeptide functions in a circadian clockwork.

### Wavelet Transform–Based Method Improved Reliability of Multiscale Event Detection

Our proposed method for detection of events performed well in these recordings with events over multiple timescales and shapes. We could show events that spanned three orders of magnitude in timescale. They were well recognized and characterized, even in cases where they overlapped at multiple scales. In comparison to other existing methods for event detection in electrophysiology recordings in the literature (Guzman et al., 2014; Merel et al., 2016; Pernía-Andrade et al., 2012; Rey et al., 2015; Richardson & Silberberg, 2008; Shi et al., 2010), this method required fewer restrictions and assumptions concerning the duration of events. Our proposed method only requires defining maximum and minimum timescales, and the intermediate scales will be spanned automatically. Methods that apply a threshold over a filtered version of the original recording are very popular in spike detection. Often, the estimation of a suitable theshold is based on the assumption that spikes are very short, compared with the interspike intervals. This condition is not fulfilled in signals containing synaptic events. For those cases, threshold methods are outperformed by techniques based on template matching (Shi et al., 2010) and, more recently, on deconvolution of the signals (Guzman et al., 2014; Merel et al., 2016; Pernía-Andrade et al., 2012). Deconvolution methods can be seen as more sophisticated versions of template matching (Merel et al., 2016). Both series of methods required the previous extraction of representative samples from the trace (Merel et al., 2016; Pernía-Andrade et al., 2012). While these approaches work well when the shapes are consistent over the whole recording, they fail for multiscale, variable events (Merel et al., 2016). Widely differing durations of events are prone to cause many false positives and negatives (see Supporting Information). Our approach avoids these problems by not using extracted templates from the trace, and selectively highlighting the duration of the events. In addition, a greater robustness against noise arises. Coherent with our own tests, deconvolution methods perform well under the presence of low to moderate noise in the signal (deconvolution methods have been reported to improve detection with signal-to-noise (SNR) = 5, Pernía-Andrade et al. (2012). In contrast, the method presented here is able to detect the signature of each event even under noise of higher amplitude (SNR = 1 and SNR = 0.5).

### Activity Patterns of the Cockroach Clock Were Typical for Properties of Coupled Endogenous Oscillators

As a result of our exploratory analysis of activity intensity heatmaps (Figures 5–7) and cluster analysis (Figure S1), we found events and episodes that provided the basis for modeling approaches as well as for experiments to test our hypothesis of neuropeptide actions. Phenomena that were described in other dynamical systems, such as intermittency (Stavrinides & Anagnostopoulos, 2013; Żebrowski & Baranowski, 2004), are fundamental in the process of resolving the circuit topology in the cockroach clock. The onset of large-amplitude oscillations observed in the cockroach clock (Figure 6F) was reminiscent of the passage through Hopf bifurcations under slow changes of parameters in the underlying system (Baer et al., 1989). Concurrent amplitude modulation of events at well-separated timescales observed in the cockroach clock (10 and 10 min; see Figure 7B) resembled a self-similar structure. Self-similar structures (“fractals”) occur in complex networks (Gallos, Makse, & Sigman, 2012), near bifurcations (Kwok & Smith, 2005), and also as a result of neuronal avalanches (Gireesh & Plenz, 2008). In our modeling of the cockroach clock we currently combine the construction of minimal oscillator networks that reproduce the observed physiological features along the lines of previous publications (Izhikevich, 2007; Tokuda et al., 2015) and that are in accordance with known neuroanatomical circuit properties (reviews: Stengl & Arendt, 2016).

### Alpha, Beta, and Gamma Frequency Band Oscillations Occur in Mammalian and Insect Brains Alike

Frequency bands in the alpha range of 8 to 12 Hz were first detected in the 1920s in extracellular recordings of the cortex, in human electroencephalograms (EEGs; Berger, 1929). Since then, different frequency bands from 0.05 to 500 Hz were described for the mammalian brain that were associated respectively with specific cognitive functions (reviews: Buzsáki, 2015; Buzsáki & Draguhn, 2004; Engel, Fries, & Singer, 2001). Recent work described faster oscillations in the rat clock (SCN) *in vivo* (Tsuji, Tsuji, Ludwig, & Leng, 2016) or in cell culture (Kononenko, Honma, & Honma, 2013). Fundamental frequencies of 32 Hz were found in rat circadian clock neurons that responded to the onset or the offset of a light stimulus given to the eye (Tsuji et al., 2016). This fundamental gamma frequency was present in the rat circadian clock throughout the day. It is currently unclear how these ultradian rhythms contribute to a circadian rhythmicity. However, they seemed to be tightly linked to environmental light input (Belle & Diekman, 2018), which is congruent with our findings. In general, smaller compact networks with fewer neurons oscillated at higher frequencies and lower amplitudes, while very large spatially distributed networks with synchronized activity of many neurons produced slower oscillations at larger amplitudes (Buzsáki & Draguhn, 2004; Csicsvari, Jamieson, Wise, & Buzsáki, 2003; Steriade, 2001). Thus, highly developed features of connectivity in a light-dependent mammalian clock, as well as more general features of connectivity, affected amplitude and frequency of oscillations at different frequency bands in human/mammalian brains. Consequently, the question arose whether oscillations at different frequency bands can be observed only in the complex mammalian brain as signature of its unique, highly evolved functional connectivity. Alternatively, this could be an evolutionary old property of neural networks serving specific functions that humans share with animals of different species. In line with this hypothesis, gamma oscillations in the central brain of *Drosophila melanogaster* were found to be evoked by olfactory stimulation (Paulk, Zhou, Stratton, Liu, & van Swinderen, 2013). Electroretinograms recorded from the optic lobe of the blow fly revealed a double-frequency peak around 150 Hz that corresponds to high gamma frequencies (Kirschfeld, 1992), described also in the visual cortex of monkeys (∼ 70–80 Hz, high gamma; Ray & Maunsell, 2010, 2011; Van Kerkoerle et al., 2014; Womelsdorf, Fries, Mitra, & Desimone, 2006). Since gamma band frequencies found in mammalian brains also occurred in our electrophysiological recordings of the cockroach clock, this suggests that network oscillations as gamma frequency oscillations are a general property of neural networks indicative of shared principles of connectivity between species. Integrating faster neural oscillations (alpha, beta, gamma) into models of the circadian clock is still quite unexplored. As the cockroach clock is relatively less complex and comprises a much smaller number of neurons in comparison to the mammalian clock, exploring the interplay between faster neural oscillations and the circadian rhythm in the cockroach clock via experiments and modeling might provide more insights into underlying mechanisms.

### Insect Clock Neurons Generate Circadian Outputs Apparently via Beta and Gamma Band Ensemble Formation to Control Sleep-Wake Cycles

**Figure 9.**
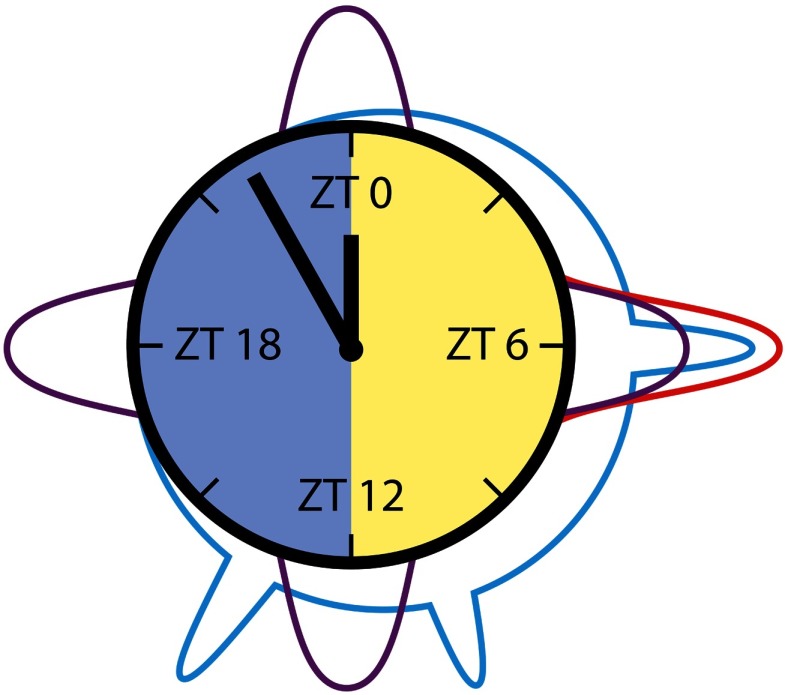
Cartoon summary of main results: Beta/gamma, beta, and alpha frequencies prevailed at different Zeitgeber times (ZTs) in long-term recordings of the cockroach clock. Beta/gamma frequencies (20–40 Hz, cyan) peaked around midday, evening, and early night. They are hypothesized to be related to PDF release. Beta frequencies (12–20 Hz, purple) with 6-hr periodicity were suggested to be based upon clock-dependent regular neuropeptide release. Alpha frequencies (8–Hz, red), which were dominantly present around midday, occurred during the cockroaches’ sleep. Next, we will examine whether there are causal relationships between prevailing frequencies, neuropeptide release, and sleep-wake phases.

In this study, oscillations in the alpha range (8–12 Hz), lower beta range (12–20 Hz), and beta and gamma band range (20–40 Hz) exhibited distinctly different prevalence patterns (Figure 9). Oscillations in the alpha range (8–12 Hz) peaked sharply around midday only. In mammals, alpha oscillations were suggested to be associated with mutual inhibition (Klimesch, 2012; Tsuji et al., 2016), since the amplitude was decreased rather than increased in response to a stimulus. Inhibitory GABAergic (GABA: gamma-aminobutric acid) networks play a significant role in forming synchronized ensembles in the cockroach clock (review: Stengl et al., 2015). Furthermore, GABA was suggested to be sleep-promoting in insects (Helfrich-Förster, 2018). Prevalence of inhibitory GABA activity during the day would be congruent with the sleep-wake phases of the nocturnal cockroach (Giese et al., 2018). However, whether alpha oscillations are linked to GABA-dependent sleep-promotion in the clock network of the cockroach remains to be investigated. Oscillations in the lower beta band range (12–20 Hz) exhibited a 6-hr periodic rhythm. This is congruent with extracellular recordings of an isolated accessory medulla (AME), which revealed maximal changes of electrical activity at dawn and dusk as well during midday and more prominently around midnight (Schneider & Stengl, 2007) with a 6-hr periodicity. Since many other neuropeptides besides PDF are expressed in AME neurons, these peaks could be associated with neuropeptide release via different neuronal ensembles structuring sleep-wake cycles (Stengl & Arendt, 2016). Oscillations in the beta and gamma band range (20–40 Hz) of the cockroach circadian clock were predominantly present during the day and at dusk, correlating with the suggested time of PDF release by the AME, the insect circadian clock (reviews: Hermann-Luibl & Helfrich-Foerster, 2015; Stengl & Arendt, 2016). Insect PDF neurons are circadian clock neurons since they express circadian clock genes and innervate the AME (Helfrich-Förster, 1995; Petri, Stengl, Würden, & Homberg, 1995). Controlled via their endogenous circadian clock, they rhythmically release their neuropeptide PDF during the day, as their light-like PDF-dependent phase response curve suggests (Eck, Helfrich-Förster, & Rieger, 2016; Park et al., 2000; Petri & Stengl, 1997; Schulze, Schendzielorz, Neupert, Predel, & Stengl, 2013). In the Madeira cockroach it was shown that the number of PDF-expressing neurons increases during longer days and longer photoperiods, thus light enhanced PDF synthesis (Wei & Stengl, 2011). Furthermore, in the fruitfly *Drosophila melanogaster* PDF neurons are activated light-dependently and mediate arousal and sleep (Chatterjee et al., 2018; Shang, Griffith, & Rosbash, 2008; Sheeba, Fogle, et al., 2008; Sheeba, Gu, Sharma, O’Dowd, & Holmes, 2008). Also in the night-active Madeira cockroach PDF neurons control sleep-wake cycles. During the day, they were suggested to activate sleep-promoting neuronal circuits and inhibit arousal-promoting circuits via PDF release (Gestrich et al., 2018, review: Stengl & Arendt, 2016). Accordingly, PDF application to an AME in vitro recruited a PDF-dependent neuronal ensemble that fired synchronously in the beta/gamma frequency range (Schneider & Stengl, 2005). Further *in vivo* experiments will test whether, indeed, timed neuropeptide release by the cockroach circadian clock generates ensembles of neurons firing at specific frequencies in beta and gamma frequency bands and whether alpha frequency bands could be caused by synchronized activity of inhibitory networks. In future studies, we will challenge our hypothesis experimentally combined with modeling that the beta/gamma band prevalence during day and dusk is due to PDF release, controlling circadian sleep-wake cycles.

## MATERIALS AND METHODS

### Animals and Surgical Procedure

For all experiments, male Madeira cockroaches (*Rhyparobia maderae*) were collected from laboratory colonies kept in large plastic containers. Conditions were kept constant with a temperature of approximately 26°C, 60% relative humidity, and a constant light-dark cycle of 12:12 hr. Cockroaches were fed with dry dog food pellets, potatoes, and fruits, with water provided ad libitum.

For surgical preparation the animal was briefly anaesthetized on ice and inserted into a custom-built holder. The head was fixed with wax and thorax, and the abdomen and legs were fixed with tape. The head capsule was opened with four cuts: two cuts between both antennae and two perpendicular cuts. The head capsule was rinsed with cockroach ringer solution (NaCl 156 mM, KCl 4 mM, CaCl^2^ 1 mM, HEPES 10 mM, glucose 5 mM; pH = 7.1; 380 mOsm) before removing glands, large tracheal sacs, and fat bodies to expose the optic lobe of the brain. The neurilemma was removed above the accessory medulla (AME) (recording site) with fine forceps The location of the AME could be identified by a characteristic trachea on the brain surface (Petri & Stengl, 1997).

### Electrophysiology

Extracellular loose-patch recordings in the current clamp mode from AME neurons were performed over 24–48 hr on a vibration-free table, with a MultiClamp 700B with Digidata 1550A1 (Axon Instruments, Union City, CA, USA) under a Zeiss W N-Achroplan NA 1.0 microscope. Micromanipulators IVM-3000, Scientifica, UK, were used; additionally, in a second recording setup bridge amplifiers BRAMP-01-R and BA-03X NPI, Tamm, Germany, with CED 1401micro, Cambridge Electronic Design, Cambridge, UK, were employed. Glass electrodes (GCF 150-7.5, Harvard Apparatus, Holliston, MA, USA) were prepared with a micropipette puller (Flaming/Brown P-87, Sutter Instruments, Novato, CA, USA) and filled with 1 M KCl (Sigma) with resistances of 8–12 MΩ. After recordings, the cell membrane was electrically permeabilized and neurons were labeled by iontophoretic injection of neurobiotin with depolarizing current (2–6 nA for 1–20 min). Depending on the respective seal resistance (∼ 1 GΩ, loose-patch clamp recordings allowed to record either action potentials of single neurons (1- to 2-ms durations), or multiunit action potential activity (∼2- to 9-ms durations) of synchronized neuronal populations, or slow field potentials indicative of synchronized excitatory/inhibitory postsynaptic potentials (∼100- to 200-ms durations). All recordings in this study were performed with a seal resistance of ∼ 1 GΩ. Long-term recordings over more than 24 hr were very challenging and took a considerable amount of time to be accomplished. We only included long-term recordings in our analysis that were stable for at least 24 hr, showing stable seal resistances, no drastic drifts of the baseline, and no mechanical drift of the electrodes. Seal resistance was checked at the beginning and at the end of the recordings via current injections. Mechanical drift of the electrodes was checked under the microscope and were avoided with optimization of the setup removing any mechanical load on the micromanipulators or electrodes. Furthermore, stability of the *in vivo* recording could only be obtained with optimal fixation of the cockroach and with minimalizing the invasiveness of the operation. Finally, potential changes were monitored via an audio amplifier (12 W, Kemo Electronics, Germany) to monitor quality of the recordings acoustically. For data acquisition pClamp10 software (Axon Instruments) was employed and data were imported into Spike2 software (versions 7 or 9, CED, Cambridge, UK) for analysis. Signals were digitized and sampled at frequencies of 25–50 kHz. Aliasing was avoided according to the Nyquist theorem (Nyquist, 1928). Unfiltered recorded data were filtered respectively afterwards during data analysis to avoid filter artefacts during the recordings. High-pass filtering (200 Hz) eliminated electrode offset and low-pass filtering (2,000 Hz) reduced high-frequency noise, if necessary. Three of the 18 recordings were performed by Dr. HongYing Wei.

### Data Preprocessing

Data files were imported and processed in a Python environment. Original files in their proprietary format were converted using NEO functions (Garcia et al., 2014). Time series were forward and backward filtered in three steps for noise removal and waveform preservation with filtfilt function from Scipy (Jones, Oliphant, & Peterson, 2014). As a first step, power supply interference at 50 Hz was removed using a notch filter. A low-pass filter with a cut frequency of 6,000 Hz was then applied to remove high-frequency noise. A Savitzky-Golay filter was finally used to further smooth the signal, while preserving the amplitude of the shortest peaks.

### Event Detection

Our model signal can be expressed as

$$x(t)=\overset{N}{\underset{i=1}{\sum }}\Theta_{i}(t-\tau_{i})+B(t),$$ (1)

B(t)=Ω(t)+ξ(t),

where *x*(*t*) is the signal, Θ_*i*_(*t* − *τ*_*i*_) are the time-localized events, *B*(*t*) is the baseline of the signal, which is noisy (*ξ*(*t*)), and may contain sinusoidal oscillations (Ω(*t*)). If the waveforms of the events had a direct correspondence with the source processes, which they originated from, and if the number of sources was small in comparison to the number of events, we might find that the events Θ_*i*_(*t* − *τ*) comply with

$$\Theta_{i}(t)\in \{\Theta_{R}(t),\Theta_{1}(t),\Theta_{2}(t),\ldots ,\Theta_{K}(t)\},$$ (2)

where *K* is the number of sources, and Θ_*R*_ is reserved for those events that are outliers or random sources. In our approach, we used the wavelet transform to selectively highlight events of different timescales and to infer some of their properties, such as amplitude and duration. Although the continuous wavelet transform (CWT) and analytic wavelet transform (AWT) allowed a better resolution in time, we used a subset of the possibly present scales, prioritizing computational affordability for large datasets. For comparison, we attempted a detection by applying a threshold to the signal, as it is usually done in spike sorting pipelines (Rey et al., 2015).

### Threshold Detection

Detection by applying a signal threshold usually involves the following steps: (a) filter of the signal, to remove the portion of the noise that lies in a region of the spectra we consider to be out of interest; (b) estimate the noise properties, such as standard deviation, in order to (c) calculate an appropriate threshold as a multiple of this noise standard deviation to set for the signal; (d) detect peaks above this threshold. Depending on the signal-to-noise ratio (SNR), this method is known to be sensitive to threshold estimation, leading to higher rates of false positives when threshold is too low, and false negatives when threshold is too high. Under the assumption that the probability of finding an event is low compared with the rest of the signal, it is possible to estimate the noise and set the threshold (Rey et al., 2015).

$$Thresh=k\left(\frac{\text{median}(|x(t)|)}{0.6745}\right),$$ (3)

where *k* is a parameter that is usually between 3 and 5. In the case presented in Figure 3, the parameter *k* was manually tuned over intervals with relative stationarity, to values between 1 and 3.

### Wavelet Transform

Wavelet analyses have been successfully used to describe signals with both frequency and time resolution. The wavelet transform of a real-valued signal *x*(*t*) is defined as

$$W_{x}(\tau ,s)=\int_{-\infty }^{\infty }\frac{1}{s^{n}}\phi^{*}\left(\frac{t-\tau }{s}\right)x(t)dt,$$ (4)

where *W*_*x*_(*τ*, *s*) is the wavelet transform of the signal *x*(*t*), ϕ^*^(*t*) is the complex conjugate of the mother wavelet, *s* and *τ* are the scale and shift with respect to the mother wavelet, and the power *s*^*n*^ is used as a normalization. The wavelet transform is, therefore, a convolution that can be seen as projections onto shifted and rescaled versions of the mother wavelet. While *n* = 1/2 is the usual choice for normalization, it has been shown that *n* = 1 has some advantages, such us making the modulus of *W*_*x*_ proportional to amplitudes in oscillatory signals (Lilly, 2017; Lilly & Olhede, 2009). As this factor can be taken outside the integral, we make use of both approaches when necessary.

Wavelets are zero-mean, square integrable functions that comply with certain requirements, such as “admissibility” (Lilly & Olhede, 2009, 2010). A variety of wavelets have been proposed (e.g., Paul, derivative of Gaussian, Daubechies, and Morlet wavelets), and the properties of the wavelet transform differ greatly depending on the chosen wavelet. A most important difference between different families of wavelets is the way their support is distributed in the time/timescale plane, that is, the trade between time and timescale resolution, limited by the Heisenberg area of the wavelet (Lilly & Olhede, 2009, 2012; Torrence & Compo, 1998). If the wavelet is analytic, that is with support only in positive frequencies, Equation 4 is the expression for the analytic wavelet transform (Lilly & Olhede, 2010). In the general case, that is, when the wavelet is complex or real-valued, *W*_*x*_(*τ*, *s*) is the continuous wavelet transform. One should note that a real-valued CWT could be also obtained by taking the real part of the AWT.

The generalized Morse wavelets have been shown to be a wavelet family, from which several of the popular families of wavelets can be seen as special cases (Lilly & Olhede, 2012). It arises as a solution of a joint time/frequency localization problem (Lilly & Olhede, 2009; Olhede & Walden, 2002). It is defined in the frequency and time domain as follows:

$$\Phi_{\beta ,\gamma }(\omega )=\int_{-\infty }^{\infty }\phi_{\beta ,\gamma }(t)e^{-i\omega t}dt=U(\omega )a_{\beta ,\gamma }\omega^{\beta }e^{-\omega \gamma },$$ (5)

$$a_{\beta ,\gamma }=2{\left(\frac{e\gamma }{\beta }\right)}^{\frac{\beta }{\gamma }},$$

where *U*(*ω*) is the unit step, and *β* and *γ* are the parameters that control the wavelet shape. It can be proven that incrementing the value of *β* by 1 is equivalent to performing a time derivative. In this sense, the choice of *γ* defines the subfamily of wavelet that is obtained, while *β* determines the wavelet within this family (Lilly & Olhede, 2012). An interesting result is that a wavelet transform of a Morse wavelet is itself a modified Morse wavelet of the following form (Lilly, 2017):

$$\begin{array}{c} \int_{-\infty }^{\infty }\frac{1}{s}{\phi_{\beta ,\gamma }}^{*}\left(\frac{t-\tau }{s}\right)\phi_{\mu ,\gamma }\left(\frac{t}{s_{i}}\right)dt=\frac{a_{\beta ,\gamma }a_{\mu ,\gamma }}{a_{\beta +\mu ,\gamma }}\frac{{\left(\frac{s}{s_{i}}\right)}^{\beta }}{{\left({\left(\frac{s}{s_{i}}\right)}^{\gamma }+1\right)}^{\frac{\beta +\mu +1}{\gamma }}}\phi_{\beta +\mu ,\gamma }\left(\frac{\frac{\tau }{s_{i}}}{{\left({\left(\frac{s}{s_{i}}\right)}^{\gamma }+1\right)}^{\frac{1}{\gamma }}}\right)\\ \;=A_{\beta ,\mu ,\gamma }f_{\beta ,\mu ,\gamma }^{\left(1\right)}\left(\frac{s}{s_{i}}\right)\phi_{\beta +\mu ,\gamma }\left(\frac{\frac{\tau }{s_{i}}}{{\left({\left(\frac{s}{s_{i}}\right)}^{\gamma }+1\right)}^{\frac{1}{\gamma }}}\right) \end{array}$$ (6)

(for *n* = 1).

It is easy to show that for the case *n* = 1/2;

$$\begin{array}{c} \int_{-\infty }^{\infty }\frac{1}{s^{\frac{1}{2}}}{\phi_{\beta ,\gamma }}^{*}\left(\frac{t-\tau }{s}\right)\phi_{\mu ,\gamma }\left(\frac{t}{s_{i}}\right)dt=\frac{a_{\beta ,\gamma }a_{\mu ,\gamma }}{a_{\beta +\mu ,\gamma }}\frac{s_{i}^{\frac{1}{2}}{\left(\frac{s}{s_{i}}\right)}^{\beta +\frac{1}{2}}}{{\left({\left(\frac{s}{s_{i}}\right)}^{\gamma }+1\right)}^{\frac{\beta +\mu +1}{\gamma }}}\phi_{\beta +\mu ,\gamma }\left(\frac{\frac{\tau }{s_{i}}}{{\left({\left(\frac{s}{s_{i}}\right)}^{\gamma }+1\right)}^{\frac{1}{\gamma }}}\right)\\ \;=A_{\beta ,\mu ,\gamma }f_{\beta ,\mu ,\gamma }^{\left(\frac{1}{2}\right)}\left(\frac{s}{s_{i}}\right)\phi_{\beta +\mu ,\gamma }\left(\frac{\frac{\tau }{s_{i}}}{{\left({\left(\frac{s}{s_{i}}\right)}^{\gamma }+1\right)}^{\frac{1}{\gamma }}}\right) \end{array}$$ (7)

(for *n* = 1/2).

By deriving these expressions, it is straightforward to see that modulus of both Equation 6 and Equation 7 are maximized for *τ* = 0 (center of the wavelets are coincident) and *s* = *s*_*max*_, being *s*_*max*_ = *s*_*i*_ for *n* = 1/2 (i.e, the scales are coincident), and *s*_*max*_ = ${\left[\frac{\beta }{\mu +1}\right]}^{\frac{1}{\gamma }}s_{i}$ for *n* = 1 (modulus is maximized when the transformation is done with a scale slightly different from 1, for example, for *β* = 2, *μ* = 2, and *γ* = 3, *s*_*max*_ = 0.87 *s*_*i*_). Another interesting observation is that the resulting transform also has a compact support in the time/timescale plane. This is expected, since generalized Morse wavelets were originally constructed as solutions of a localization problem.

### Detection Using CWT or AWT

We first assumed that events were shaped with a waveform that closely resembles the selected Morse wavelet. To detect these events in the real-valued signal, we look for maxima and minima in the wavelet transform, which represent positive and negative excursions Θ_*i*_(*t* − *τ*_*i*_) with respect to the baseline signal *B*(*t*). When detected, properties of the events could be inferred from the values at the maxima and minima. The appearance time and timescale at each maxima and minima could be obtained following Equations 6 and 7. Timescales could then be transformed into an event duration. Amplitude could be inferred by taking into account that, for *n* = 1, the modulus of the wavelet transform was proportional to the amplitude. Furthermore, an estimation for this proportionality was given (Lilly, 2017).

Constructing the AWT or CWT might be computationally costly for long-term recordings that last for more than 24 hr. In our approach, we selected a limited number of scales from a geometric sequence, each of them representing a bandwidth of scales. Inspired in what is the usual discretization in the discrete wavelet transform, we took *s* = 2^*j*^Δ*T*, *j* = 0, 1, …, *N*_*s*_. From Equations 6 and 7 it was possible to see that the modulus of AWT or CWT decay enough between the scale where the maximum is detected and the next adjacent one, to clearly define a “winner” scale for the event, in cases where it was detected in more than one scale.

### Close and Overlapping Events

When events were separated enough from each other either in time or timescale axis, they were reflected in AWT and CWT as separated peaks that decayed to a base level before reaching the next peak. This separation was usually between three and four steps in the scale axis, or a multiple of the scale parameter in the time axis. When events got closer in the time/timescale plane, their support regions started to overlap. For moderate overlapping, it was still possible to identify two distinct events, as the maxima/minima points remained clear. When the overlapping was larger, events started to be indistinguishable in the modulus of the AWT, but still recognizable in the real CWT (Figure 4), which could be used in this case. If the events were equally signed, they sometimes were detected as a fused larger event that is better captured by a larger scale wavelet. This was a limitation because of the choice of a redundant base of wavelets, compared with methods that allow for better source separation, such as independent component analysis (ICA). In the case of opposite-signed events, it was necessary to distinguish events from spurious oscillations in the CWT because of the second peak in the real part of the wavelet. As a consequence, it was possible to set a minimum distance between events of the same scale and opposite sign, choosing the one that reflects the larger amplitude when this distance was not reached.

### Procedure for Event Detection

1.  Obtain a first set of pre-events by detecting maxima and minima in the real wavelet transform for each of the scales, using *n* = 1/2.
2.  Discard the pre-events that result from spurious oscillations, with the distance-amplitude criteria.
3.  For each of the pre-events, determine a winner scale when it is detected in more than one consecutive scale.
4.  Rescale the maxima and minima, using *n* = 1.
5.  Estimate the duration and the amplitude for each event.

### Event Clustering

In order to find groups of similar events, and estimate the set {Θ_*R*_(*t*), Θ_1_(*t*), Θ_2_(*t*), …, Θ_*K*_(*t*)} posed in Equation 2, clustering was performed using estimated widths, amplitudes, and time of each event as features. This election of features was also proposed in Lara et al. (2014) when performing data mining of EEG recordings. These features were first rescaled, in order to make them comparable in the calculation of Euclidean distances. Time localization was used to account for nonstationarity in waveforms; events whose shape may change slightly in a much slower timescale (i.e., hours) could still be recognized to be part of the same cluster. This approach could then be complemented, if necessary, with manual merging of clusters that showed similar behavior but showed separation in time and, therefore, were initially detected as separate clusters.

Because of the lengths of the recordings, a manual initial guess for the number of clusters would involve a time-consuming exploration. For that reason, we opted for methods that did not require the number of clusters as input parameters, but rather estimated them. Our tests indicated that for the amount of overlapping between clusters, their elongated shapes, and the choice of avoiding the initial guess of clusters, reasonable results could be obtained using Bayesian gaussian mixtures.

### Activity Descriptions

For exploratory visualization of the data, we created heatmaps for the activity at different timescales over time (Figures 5A–D; Figures 6A, B, D, E; Figure 8B). In bins of 100 s, the color represents the sum of the absolute values of the event amplitudes. The resulting graphs exhibited a first good visualization of the dominant scales at each point of time.

### Event Train Variability

Several metrics were developed for analyzing spike train variability. Some of these metrics are locally defined, while others are calculated over a time window (Eden & Kramer, 2010; Gabbiani & Koch, 1998; Ponce-Alvarez, Kilavik, & Riehle, 2010; Shinomoto et al., 2009). Usually, metrics were defined in a way that they increase as the variability in the interspike interval becomes larger. When the train shows the variability expected from a Poisson process, metrics should be equal to 1. If it is smaller, the train is said to be more regular, up to the limit of a constant interspike interval at the value of 0 (Gabbiani & Koch, 1998). We applied two of these metrics for analyzing some episodes of events occurring at similar timescales: coefficient of variation (*C*_*v*_) and Fano factor (*FF*). *C*_*v*_ and *FF* are defined over a time window as follows:

$$C_{v}=\frac{\text{std}(T_{i})}{\bar{T_{i}}},$$ (8)

$$FF=\frac{\text{var}(T_{i})}{\bar{T_{i}}},$$ (9)

where *T*_*i*_ are the inter-event intervals in the selected window. By displacing a window, it is possible to calculate time-resolved *C*_*v*_ and *FF*. Additionally, *FF* was calculated with variable window sizes.

### Time Series Frequency Analysis

In order to analyze frequency content of the signals over time, spectrograms were created using the function spectrogram from SciPy (Jones et al., 2014), in windows of 26.1 s. Frequency prevalence in the 20- to 40-Hz band, 12- to 20-Hz band, and 8- to 12-Hz band was calculated with the following procedure: (a) columns in the spectrogram were averaged in bins of 10 min, resulting in a periodogram for the bin; (b) for each periodogram, an exponential function was fitted to obtain a baseline that is representative of the background noise; (c) a background noise level was estimated as the median of the absolute values of the difference between the spectra and the baseline; (d) a background area was estimated, as the product between the total frequency range and the background noise level; (e) band area was calculated as the area of the periodogram that lies above baseline in the 20- to 40-Hz, 12- to 20-Hz, or 8- to 12-Hz bands; (f) prevalence was calculated as the ratio between band area and background area.

### Statistics

Statistically significant peaks in the prevalence of 20- to 40-Hz, 12- to 20-Hz, or 8- to 12-Hz frequencies was detected using R v. 3.6.0 and R Studio v. 1.2.1335 with a custom-written script. Data were fitted to linear mixed models with Zeitgeber time (ZT) as fixed effect and individual as random effect (lme function, nlme package; Pinheiro, Bates, DebRoy, Sarkar, & R Core Team, 2019). Periodicity of 12- to 20-Hz prevalence was tested using an ANOVA and sine fits.

## ACKNOWLEDGMENTS

We thank Dr. HongYing Wei, at the Department of Biology, Animal Physiology, of the University of Kassel for performing three of the 18 long-term *in vivo* recordings. We thank André Arand for expert animal care raising the cockroaches, Dr. Achim Werckenthin for graphical contributions, and Dr. Susanne Neupert, Dr. Paula Kuokkanen, and Dr. Claudia Arbeitman for their valuable comments.

## SUPPORTING INFORMATION

Supporting Information for this article is available at https://doi.org/10.1162/netn_a_00106.

## ETHICAL APPROVAL

All animal procedures were in compliance with the guidelines of the European Union (Directive 2010/63/EU) and the German Animal Welfare Act.

## DATA ACCESSIBILITY STATEMENT

All raw data and statistical analyses will be made accessible upon request.

## AUTHOR CONTRIBUTIONS

Pablo Rojas: Data curation; Formal analysis; Methodology; Software; Validation; Visualization; Writing – Original Draft; Writing – Review & Editing. Jenny Plath: Visualization; Formal analysis; Writing – Review & Editing. Julia Gestrich: Data curation; Methodology. Bharath Ananthasubramaniam: Formal analysis; Methodology; Writing – Review & Editing. Martin Garcia: Formal analysis; Methodology; Project administration; Resources; Supervision; Writing – Review & Editing. Hanspeter Herzel: Funding acquisition; Methodology; Project administration; Resources; Supervision; Writing – Review & Editing. Monika Stengl: Conceptualization; Funding acquisition; Project administration; Resources; Supervision; Writing – Original Draft; Writing – Review & Editing.

## FUNDING INFORMATION

Monika Stengl, Deutsche Forschungsgemeinschaft (http://dx.doi.org/10.13039/501100001659), Award ID: STE531/26-1; SPP 2041. Monika Stengl, Deutsche Forschungsgemeinschaft (http://dx.doi.org/10.13039/501100001659), Award ID: STE531/18-2. Monika Stengl, Deutsche Forschungsgemeinschaft (http://dx.doi.org/10.13039/501100001659), Award ID: STE531/ 18-3. Hanspeter Herzel, Deutsche Forschungsgemeinschaft (http://dx.doi.org/10.13039/501100001659), Award ID: HE2168/11-1; SPP 2041. Monika Stengl, University of Kassel (http://dx.doi.org/10.13039/501100012687), Award ID: Biological clocks, graduate school program.

## TECHNICAL TERMS

Accessory medulla: A small neuropil in the optic lobe of the insect brain that is innervated by processes of clock neurons.
PDF (pigment-dispersing factor): Evolutionary conserved neuropeptide that synchronizes insect clock cells.
Zeitgeber time: Rhythm provided by an external environmental cue, which is able to entrain biological clocks.
Wavelet transform: Decomposition of a signal in a set of scaled and shifted wave-like functions called wavelets.
Fano factor: Metric used for variability in interevent intervals, with a Poisson distribution showing a Fano factor equal to 1.
Bifurcation: A sudden transition in the qualitative behavior of a system because of small and smooth changes in its parameters.
Intermittency: Intermittent transition between regular behavior and irregular bursts.
Hopf bifurcation: A bifurcation where sustained oscillations arise because of a change in the stability of an equilibrium point.
Loose-patch recording: An extracellular current clamp (or voltage clamp) recording with a patch electrode that is attached to the cell with low seal resistance.

## References

[bib1] Baer S. M., Erneux T., & Rinzel J. (1989). The slow passage through a Hopf bifurcation: Delay, memory effects, and resonance. SIAM Journal on Applied mathematics, 49(1), 55–71.

[bib2] Belle M. D., & Diekman C. O. (2018). Neuronal oscillations on an ultra-slow timescale: Daily rhythms in electrical activity and gene expression in the mammalian master circadian clockwork. European Journal of Neuroscience, 48(8), 2696–2717. 29396876 10.1111/ejn.13856

[bib3] Berger H. (1929). Über das elektrenkephalogramm des menschen. European Archives of Psychiatry and Clinical Neuroscience, 87(1), 527–570.

[bib4] Buzsáki G. (2015). Hippocampal sharp wave-ripple: A cognitive biomarker for episodic memory and planning. Hippocampus, 25(10), 1073–1188. 26135716 10.1002/hipo.22488 PMC4648295

[bib5] Buzsáki G., & Draguhn A. (2004). Neuronal oscillations in cortical networks. Science, 304(5679), 1926–1929. 15218136 10.1126/science.1099745

[bib6] Chatterjee A., Lamaze A., De J., Mena W., Chélot E., Martin B., … Rouyer F. (2018). Reconfiguration of a multi-oscillator network by light in the *Drosophila* circadian clock. Current Biology, 28(13), 2007–2017. 29910074 10.1016/j.cub.2018.04.064 PMC6039274

[bib7] Csicsvari J., Jamieson B., Wise K. D., & Buzsáki G. (2003). Mechanisms of gamma oscillations in the hippocampus of the behaving rat. Neuron, 37(2), 311–322. 12546825 10.1016/s0896-6273(02)01169-8

[bib8] Eck S., Helfrich-Förster C., & Rieger D. (2016). The timed depolarization of morning and evening oscillators phase shifts the circadian clock of *Drosophila*. Journal of Biological Rhythms, 31(5), 428–442. 27269519 10.1177/0748730416651363

[bib9] Eden U. T., & Kramer M. A. (2010). Drawing inferences from Fano factor calculations. Journal of Neuroscience Methods, 190(1), 149–152. 20416340 10.1016/j.jneumeth.2010.04.012

[bib10] Engel A. K., Fries P., & Singer W. (2001). Dynamic predictions: Oscillations and synchrony in top-down processing. Nature Reviews Neuroscience, 2(10), 704. 11584308 10.1038/35094565

[bib11] Gabbiani F., & Koch C. (1998). Principles of spike train analysis. In Methods in neuronal modeling (Vol. 12, pp. 313–360). Citeseer.

[bib12] Gallos L. K., Makse H. A., & Sigman M. (2012). A small world of weak ties provides optimal global integration of self-similar modules in functional brain networks. Proceedings of the National Academy of Sciences, 109(8), 2825–2830. 10.1073/pnas.1106612109 PMC3286928 22308319

[bib13] Garcia S., Guarino D., Jaillet F., Jennings T. R., Pröpper R., Rautenberg P. L., … Davison A. P. (2014). Neo: An object model for handling electrophysiology data in multiple formats. Frontiers in Neuroinformatics, 8, 10. 24600386 10.3389/fninf.2014.00010 PMC3930095

[bib14] Gestrich J., Giese M., Shen W., Zhang Y., Voss A., Popov C., … Wei H. (2018). Sensitivity to pigment-dispersing factor (PDF) is cell-type specific among PDF-expressing circadian clock neurons in the Madeira cockroach. Journal of Biological Rhythms, 33(1), 35–51. 29179611 10.1177/0748730417739471

[bib15] Giese M., Gestrich J., Massah A., Peterle J., Wei H., & Stengl M. (2018). GABA- and serotonin-expressing neurons take part in inhibitory as well as excitatory input pathways to the circadian clock of the Madeira cockroach *Rhyparobia maderae*. European Journal of Neuroscience, 47(9), 1067–1080. 29430734 10.1111/ejn.13863

[bib16] Gireesh E. D., & Plenz D. (2008). Neuronal avalanches organize as nested theta- and beta/gamma-oscillations during development of cortical layer 2/3. Proceedings of the National Academy of Sciences, 105(21), 7576–7581. 10.1073/pnas.0800537105 PMC2396689 18499802

[bib17] Guralnik V., & Srivastava J. (1999). Event detection from time series data. In Proceedings of the fifth ACM SIGKDD international conference on knowledge discovery and data mining (pp. 33–42).

[bib18] Guzman S. J., Schlögl A., & Schmidt-Hieber C. (2014). Stimfit: Quantifying electrophysiological data with Python. Frontiers in Neuroinformatics, 8, 16. 24600389 10.3389/fninf.2014.00016 PMC3931263

[bib19] Hatton G. I. (1982). Phasic bursting activity of rat paraventricular neurones in the absence of synaptic transmission. Journal of Physiology, 327(1), 273–284. 6288925 10.1113/jphysiol.1982.sp014231 PMC1225108

[bib20] Helfrich-Förster C. (1995). The period clock gene is expressed in central nervous system neurons which also produce a neuropeptide that reveals the projections of circadian pacemaker cells within the brain of *Drosophila melanogaster*. Proceedings of the National Academy of Sciences, 92(2), 612–616. 10.1073/pnas.92.2.612 PMC42792 7831339

[bib21] Helfrich-Förster C. (2018). Sleep in insects. Annual Review of Entomology, 63, 69–86. 10.1146/annurev-ento-020117-043201 28938081

[bib22] Hermann-Luibl C., & Helfrich-Foerster C. (2015). Clock network in *Drosophila*. Current Opinion in Insect Science, 7, 65–70. 10.1016/j.cois.2014.11.003 32846682

[bib23] Izhikevich E. M. (2007). Dynamical systems in neuroscience. Cambridge, MA: MIT Press.

[bib24] Jones E., Oliphant T., & Peterson P. (2014). {SciPy}: Open source scientific tools for {Python}.

[bib25] Kamimoto S., Nohara R., & Ichikawa T. (2006). Coordination between the electrical activity of developing indirect flight muscles and the firing activity of a population of neurosecretory cells in the silkmoth, *Bombyx mori*. Zoological Science, 23(5), 449–458. 16766864 10.2108/zsj.23.449

[bib26] Khanna A., Pascual-Leone A., Michel C. M., & Farzan F. (2015). Microstates in resting-state EEG: Current status and future directions. Neuroscience and Biobehavioral Reviews, 49, 105–113. 25526823 10.1016/j.neubiorev.2014.12.010 PMC4305485

[bib27] Kirschfeld K. (1992). Oscillations in the insect brain: Do they correspond to the cortical gamma-waves of vertebrates? Proceedings of the National Academy of Sciences, 89(10), 4764–4768. 10.1073/pnas.89.10.4764 PMC49164 1584816

[bib28] Klimesch W. (2012). Alpha-band oscillations, attention, and controlled access to stored information. Trends in Cognitive Sciences, 16(12), 606–617. 23141428 10.1016/j.tics.2012.10.007 PMC3507158

[bib29] Kononenko N. I., Honma S., & Honma K.-I. (2013). Fast synchronous oscillations of firing rate in cultured rat suprachiasmatic nucleus neurons: Possible role in circadian synchronization in the intact nucleus. Neuroscience Research, 75(3), 218–227. 23415823 10.1016/j.neures.2013.01.003

[bib30] Kwok T., & Smith K. A. (2005). Optimization via intermittency with a self-organizing neural network. Neural Computation, 17(11), 2454–2481. 16156935 10.1162/0899766054796860

[bib31] Lara J. A., Lizcano D., Pérez A., & Valente J. P. (2014). A general framework for time series data mining based on event analysis: Application to the medical domains of electroencephalography and stabilometry. Journal of Biomedical Informatics, 51, 219–241. 24948199 10.1016/j.jbi.2014.06.003

[bib32] Leise T. L. (2013). Wavelet analysis of circadian and ultradian behavioral rhythms. Journal of Circadian Rhythms, 11(1), 5. 23816159 10.1186/1740-3391-11-5 PMC3717080

[bib33] Lilly J. M. (2017). Element analysis: A wavelet-based method for analysing time-localized events in noisy time series. Proceedings of the Royal Society A: Mathematical, Physical and Engineering Sciences, 473(2200), 20160776. 10.1098/rspa.2016.0776 PMC5415685 28484325

[bib34] Lilly J. M., & Olhede S. C. (2009). Higher-order properties of analytic wavelets. IEEE Transactions on Signal Processing, 57(1), 146–160.

[bib35] Lilly J. M., & Olhede S. C. (2010). On the analytic wavelet transform. IEEE Transactions on Information Theory, 56(8), 4135–4156.

[bib36] Lilly J. M., & Olhede S. C. (2012). Generalized Morse wavelets as a superfamily of analytic wavelets. IEEE Transactions on Signal Processing, 60(11), 6036–6041.

[bib37] Loesel R., & Homberg U. (2001). Anatomy and physiology of neurons with processes in the accessory medulla of the cockroach *Leucophaea maderae*. Journal of Comparative Neurology, 439(2), 193–207. 11596048 10.1002/cne.1342

[bib38] Masimore B., Kakalios J., & Redish A. (2004). Measuring fundamental frequencies in local field potentials. Journal of Neuroscience Methods, 138(1–2), 97–105. 15325117 10.1016/j.jneumeth.2004.03.014

[bib39] Merel J., Shababo B., Naka A., Adesnik H., & Paninski L. (2016). Bayesian methods for event analysis of intracellular currents. Journal of Neuroscience Methods, 269, 21–32. 27208694 10.1016/j.jneumeth.2016.05.015

[bib40] Nishiitsutsuji-Uwo J., & Pittendrigh C. S. (1968). Central nervous system control of circadian rhythmicity in the cockroach. Zeitschrift für vergleichende Physiologie, 58(1), 1–13.

[bib41] Nyquist H. (1928). Certain topics in telegraph transmission theory. Transactions of the American Institute of Electrical Engineers, 47(2), 617–644.

[bib42] Olhede S. C., & Walden A. T. (2002). Generalized Morse wavelets. IEEE Transactions on Signal Processing, 50(11), 2661–2670.

[bib43] Page T. L. (1984). Neuronal organization of a circadian clock in the cockroach *Leucophaea maderae*. Photoperiodic Regulation of Insect and Molluscan Hormones, 104, 115–131. 6562958

[bib44] Park J. H., Helfrich-Förster C., Lee G., Liu L., Rosbash M., & Hall J. C. (2000). Differential regulation of circadian pacemaker output by separate clock genes in *Drosophila*. Proceedings of the National Academy of Sciences, 97(7), 3608–3613. 10.1073/pnas.070036197 PMC16287 10725392

[bib45] Patton A. P., & Hastings M. H. (2018). The suprachiasmatic nucleus. Current Biology, 28(15), R816–R822. 30086310 10.1016/j.cub.2018.06.052

[bib46] Paulk A. C., Zhou Y., Stratton P., Liu L., & van Swinderen B. (2013). Multichannel brain recordings in behaving *Drosophila* reveal oscillatory activity and local coherence in response to sensory stimulation and circuit activation. Journal of Neurophysiology, 110(7), 1703–1721. 23864378 10.1152/jn.00414.2013 PMC4868374

[bib47] Pernía-Andrade A. J., Goswami S. P., Stickler Y., Fröbe U., Schlögl A., & Jonas P. (2012). A deconvolution-based method with high sensitivity and temporal resolution for detection of spontaneous synaptic currents in vitro and in vivo. Biophysical Journal, 103(7), 1429–1439. 23062335 10.1016/j.bpj.2012.08.039 PMC3471482

[bib48] Petri B., & Stengl M. (1997). Pigment-dispersing hormone shifts the phase of the circadian pacemaker of the cockroach *Leucophaea maderae*. Journal of Neuroscience, 17(11), 4087–4093. 9151725 10.1523/JNEUROSCI.17-11-04087.1997 PMC6573569

[bib49] Petri B., Stengl M., Würden S., & Homberg U. (1995). Immunocytochemical characterization of the accessory medulla in the cockroach *Leucophaea maderae*. Cell and Tissue Research, 282(1), 3–19. 8581923 10.1007/BF00319128

[bib50] Pinheiro J., Bates D., DebRoy S., Sarkar D., & R Core Team (2019). nlme: Linear and nonlinear mixed effects models [Computer software manual]. Retrieved from https://CRAN.R-project.org/package=nlme (R package version 3.1-140)

[bib51] Ponce-Alvarez A., Kilavik B. E., & Riehle A. (2010). Comparison of local measures of spike time irregularity and relating variability to firing rate in motor cortical neurons. Journal of Computational Neuroscience, 29(1–2), 351–365. 19449094 10.1007/s10827-009-0158-2

[bib52] Principe J. C., & Brockmeier A. J. (2015). Representing and decomposing neural potential signals. Current Opinion in Neurobiology, 31, 13–17. 25113153 10.1016/j.conb.2014.07.023

[bib53] Ray S., & Maunsell J. H. (2010). Differences in gamma frequencies across visual cortex restrict their possible use in computation. Neuron, 67(5), 885–896. 20826318 10.1016/j.neuron.2010.08.004 PMC3001273

[bib54] Ray S., & Maunsell J. H. (2011). Different origins of gamma rhythm and high-gamma activity in macaque visual cortex. PLoS Biology, 9(4), e1000610. 21532743 10.1371/journal.pbio.1000610 PMC3075230

[bib55] Reischig T., & Stengl M. (2003a). Ectopic transplantation of the accessory medulla restores circadian locomotor rhythms in arrhythmic cockroaches (*Leucophaea maderae*). Journal of Experimental Biology, 206(11), 1877–1886. 12728009 10.1242/jeb.00373

[bib56] Reischig T., & Stengl M. (2003b). Ultrastructure of pigment-dispersing hormone-immunoreactive neurons in a three-dimensional model of the accessory medulla of the cockroach *Leucophaea maderae*. Cell and Tissue Research, 314(3), 421–435. 14557869 10.1007/s00441-003-0772-7

[bib57] Rey H. G., Pedreira C., & Quiroga R. Q. (2015). Past, present and future of spike sorting techniques. Brain Research Bulletin, 119, 106–117. 25931392 10.1016/j.brainresbull.2015.04.007 PMC4674014

[bib58] Richardson M. J., & Silberberg G. (2008). Measurement and analysis of postsynaptic potentials using a novel voltage-deconvolution method. Journal of Neurophysiology, 99(2), 1020–1031. 18046003 10.1152/jn.00942.2007

[bib59] Schneider N.-L., & Stengl M. (2005). Pigment-dispersing factor and GABA synchronize cells of the isolated circadian clock of the cockroach *Leucophaea maderae*. Journal of Neuroscience, 25(21), 5138–5147. 15917454 10.1523/JNEUROSCI.5138-A-04.2005 PMC6724822

[bib60] Schneider N.-L., & Stengl M. (2007). Extracellular long-term recordings of the isolated accessory medulla, the circadian pacemaker center of the cockroach *Leucophaea maderae*, reveal ultradian and hint circadian rhythms. Journal of Comparative Physiology A, 193(1), 35–42. 10.1007/s00359-006-0169-7 16983545

[bib61] Schulze J., Schendzielorz T., Neupert S., Predel R., & Stengl M. (2013). Neuropeptidergic input pathways to the circadian pacemaker center of the madeira cockroach analysed with an improved injection technique. European Journal of Neuroscience, 38(6), 2842–2852. 23802608 10.1111/ejn.12285

[bib62] Shang Y., Griffith L. C., & Rosbash M. (2008). Light-arousal and circadian photoreception circuits intersect at the large PDF cells of the *Drosophila* brain. Proceedings of the National Academy of Sciences, 105(50), 19587–19594. 10.1073/pnas.0809577105 PMC2596742 19060186

[bib63] Sheeba V., Fogle K. J., Kaneko M., Rashid S., Chou Y.-T., Sharma V. K., & Holmes T. C. (2008). Large ventral lateral neurons modulate arousal and sleep in *Drosophila*. Current Biology, 18(20), 1537–1545. 18771923 10.1016/j.cub.2008.08.033 PMC2597195

[bib64] Sheeba V., Gu H., Sharma V. K., O’Dowd D. K., & Holmes T. C. (2008). Circadian- and light-dependent regulation of resting membrane potential and spontaneous action potential firing of *Drosophila* circadian pacemaker neurons. Journal of Neurophysiology, 99(2), 976–988. 18077664 10.1152/jn.00930.2007 PMC2692874

[bib65] Shi Y., Nenadic Z., & Xu X. (2010). Novel use of matched filtering for synaptic event detection and extraction. PLoS ONE, 5(11), e15517. 21124805 10.1371/journal.pone.0015517 PMC2991367

[bib66] Shinomoto S., Kim H., Shimokawa T., Matsuno N., Funahashi S., Shima K., … Toyama K. (2009). Relating neuronal firing patterns to functional differentiation of cerebral cortex. PLoS Computational Biology, 5(7), e1000433. 19593378 10.1371/journal.pcbi.1000433 PMC2701610

[bib67] Stavrinides S. G., & Anagnostopoulos A. N. (2013). The route from synchronization to desynchronization of chaotic operating circuits and systems. In Applications of chaos and nonlinear dynamics in science and engineering (Vol. 3, pp. 229–275). Berlin, Germany: Springer.

[bib68] Stengl M., & Arendt A. (2016). Peptidergic circadian clock circuits in the Madeira cockroach. Current Opinion in Neurobiology, 41, 44–52. 27575405 10.1016/j.conb.2016.07.010

[bib69] Stengl M., & Homberg U. (1994). Pigment-dispersing hormone-immunoreactive neurons in the cockroach *Leucophaea maderae* share properties with circadian pacemaker neurons. Journal of Comparative Physiology A, 175(2), 203–213. 10.1007/BF00215116 8071895

[bib70] Stengl M., Werckenthin A., & Wei H. (2015). How does the circadian clock tick in the Madeira cockroach? Current Opinion in Insect Science, 12, 38–45.

[bib71] Steriade M. (2001). Impact of network activities on neuronal properties in corticothalamic systems. Journal of Neurophysiology, 86(1), 1–39. 11431485 10.1152/jn.2001.86.1.1

[bib72] Tokuda I. T., Ono D., Ananthasubramaniam B., Honma S., Honma K.-I., & Herzel H. (2015). Coupling controls the synchrony of clock cells in development and knockouts. Biophysical Journal, 109(10), 2159–2170. 26588574 10.1016/j.bpj.2015.09.024 PMC4656860

[bib73] Torrence C., & Compo G. P. (1998). A practical guide to wavelet analysis. Bulletin of the American Meteorological Society, 79(1), 61–78.

[bib74] Tsuji T., Tsuji C., Ludwig M., & Leng G. (2016). The rat suprachiasmatic nucleus: the Master clock ticks at 30 Hz. Journal of Physiology, 594(13), 3629–3650. 27061101 10.1113/JP272331 PMC4929337

[bib75] Tu C.-L., Hwang W.-L., & Ho J. (2005). Analysis of singularities from modulus maxima of complex wavelets. IEEE Transactions on Information Theory, 51(3), 1049–1062.

[bib76] Van Kerkoerle T., Self M. W., Dagnino B., Gariel-Mathis M.-A., Poort J., Van Der Togt C., & Roelfsema P. R. (2014). Alpha and gamma oscillations characterize feedback and feedforward processing in monkey visual cortex. Proceedings of the National Academy of Sciences, 111(40), 14332–14341. 10.1073/pnas.1402773111 PMC4210002 25205811

[bib77] Vansteensel M. J., Michel S., & Meijer J. H. (2008). Organization of cell and tissue circadian pacemakers: A comparison among species. Brain Research Reviews, 58(1), 18–47. 18061682 10.1016/j.brainresrev.2007.10.009

[bib78] Vosko A. M., Schroeder A., Loh D. H., & Colwell C. S. (2007). Vasoactive intestinal peptide and the mammalian circadian system. General and Comparative Endocrinology, 152(2–3), 165–175. 17572414 10.1016/j.ygcen.2007.04.018 PMC1994114

[bib79] Wei H., el Jundi B., Homberg U., & Stengl M. (2010). Implementation of pigment-dispersing factor-immunoreactive neurons in a standardized atlas of the brain of the cockroach *Leucophaea maderae*. Journal of Comparative Neurology, 518(20), 4113–4133. 20878779 10.1002/cne.22471

[bib80] Wei H., & Stengl M. (2011). Light affects the branching pattern of peptidergic circadian pacemaker neurons in the brain of the cockroach *Leucophaea maderae*. Journal of Biological Rhythms, 26(6), 507–517. 22215609 10.1177/0748730411419968

[bib81] Wei H., Yasar H., Funk N. W., Giese M., Baz E.-S., & Stengl M. (2014). Signaling of pigment-dispersing factor (PDF) in the Madeira cockroach *Rhyparobia maderae*. PLoS ONE, 9(9), e108757. 25269074 10.1371/journal.pone.0108757 PMC4182629

[bib82] Womelsdorf T., Fries P., Mitra P. P., & Desimone R. (2006). Gamma-band synchronization in visual cortex predicts speed of change detection. Nature, 439(7077), 733. 16372022 10.1038/nature04258

[bib83] Żebrowski J., & Baranowski R. (2004). Type I intermittency in nonstationary systems—Models and human heart rate variability. Physica A: Statistical Mechanics and Its Applications, 336(1–2), 74–83.

