# Supplementary material for: Beyond spikes: Multiscale computational analysis of in vivo long-term recordings in the cockroach circadian clock
Source: Netw Neurosci. 2019 Sep 1;3(4):944–68. doi: 10.1162/netn_a_00106 (PMC6777951; doi:10.1162/netn_a_00106)
Supplement: Supplementary file 1 [file netn-03-944-s001.pdf]

Supplementary Materials

Rojas, P., Plath, J. A., Gestrich, J., Ananthasubramaniam, B., Garcia, M. E., Herzel, H., & Stengl, M. (2019). Supporting information for "Beyond spikes: multiscale computational analysis of in vivo longterm recordings in the cockroach circadian clock." *Network Neuroscience*, *3*(4), 944–968. https://doi.org/10.1162/netn_a_00106

Table S1. Summary of Linear Mixed Model with Zeitgeber time (ZT) as fixed factor (prevalence ~ ZT, random factor = individual) tested against the lowest prevalence value at ZT 21.5 and Summary of Linear Mixed Model with time of day (TOD) as the fixed factor (prevalence ~ TOD, random factor = individual) tested against 'Day'.

		Value	Std. Error	DF	t	p
8 - 12 Hz	Intercept	0.0337	0.0106	344	3.189	0.002
	ZT0.5	0.0039	0.0149	344	0.258	0.797
	ZT1.5	0.0157	0.0155	344	1.012	0.312
	ZT2.5	0.0027	0.0155	344	0.178	0.859
	ZT3.5	-0.0004	0.0155	344	-0.025	0.980
	ZT4.5	0.0044	0.0158	344	0.280	0.780
	ZT5.5	0.0456	0.0161	344	2.827	0.005
	ZT6.5	0.0191	0.0152	344	1.257	0.210
	ZT7.5	0.0217	0.0147	344	1.475	0.141
	ZT8.5	0.0001	0.0147	344	0.004	0.997
	ZT9.5	0.0015	0.0147	344	0.101	0.920
	ZT10.5	0.0016	0.0147	344	0.106	0.916
	ZT11.5	0.0036	0.0147	344	0.247	0.805
	ZT12.5	-0.0002	0.0145	344	-0.016	0.987
	ZT13.5	0.0014	0.0145	344	0.096	0.923
	ZT14.5	-0.0009	0.0147	344	-0.059	0.953
	ZT15.5	-0.0003	0.0147	344	-0.019	0.985
	ZT16.5	0.0028	0.0147	344	0.193	0.847
	ZT17.5	0.0093	0.0147	344	0.633	0.527
	ZT18.5	0.0160	0.0147	344	1.088	0.278
	ZT19.5	0.0029	0.0147	344	0.196	0.845
	ZT20.5	-0.0001	0.0149	344	-0.004	0.997
	ZT22.5	0.0041	0.0149	344	0.275	0.784
	ZT23.5	0.0060	0.0149	344	0.402	0.688

Table S1 cont.

		Value	Std. Error	DF	t	p
12 - 20 Hz	Intercept	0.0387	0.0083	344	4.634	0.000
	ZT0.5	-0.0005	0.0118	344	-0.043	0.966
	ZT1.5	0.0040	0.0122	344	0.329	0.742
	ZT2.5	-0.0024	0.0122	344	-0.198	0.843
	ZT3.5	-0.0053	0.0122	344	-0.431	0.667
	ZT4.5	0.0020	0.0125	344	0.163	0.870
	ZT5.5	0.0030	0.0127	344	0.236	0.814
	ZT6.5	0.0218	0.0120	344	1.821	0.070
	ZT7.5	0.0079	0.0116	344	0.680	0.497
	ZT8.5	-0.0062	0.0116	344	-0.533	0.595
	ZT9.5	-0.0025	0.0116	344	-0.219	0.827
	ZT10.5	0.0031	0.0116	344	0.267	0.790
	ZT11.5	0.0109	0.0116	344	0.939	0.348
	ZT12.5	0.0202	0.0115	344	1.760	0.079
	ZT13.5	0.0011	0.0115	344	0.092	0.927
	ZT14.5	-0.0015	0.0116	344	-0.127	0.899
	ZT15.5	-0.0038	0.0116	344	-0.325	0.746
	ZT16.5	0.0010	0.0116	344	0.083	0.934
	ZT17.5	0.0025	0.0116	344	0.216	0.829
	ZT18.5	0.0080	0.0116	344	0.686	0.493
	ZT19.5	0.0003	0.0116	344	0.022	0.983
	ZT20.5	-0.0039	0.0118	344	-0.335	0.738
	ZT22.5	0.0028	0.0118	344	0.238	0.812
	ZT23.5	0.0045	0.0118	344	0.379	0.705
20 -40 Hz	Intercept	0.0332	0.0065	344	5.139	0.000
	ZT0.5	0.0073	0.0091	344	0.799	0.425
	ZT1.5	0.0055	0.0094	344	0.582	0.561
	ZT2.5	0.0142	0.0094	344	1.507	0.133
	ZT3.5	0.0138	0.0094	344	1.463	0.145
	ZT4.5	0.0105	0.0096	344	1.086	0.278
	ZT5.5	0.0129	0.0099	344	1.310	0.191
	ZT6.5	0.0251	0.0093	344	2.711	0.007
	ZT7.5	0.0152	0.0090	344	1.695	0.091
	ZT8.5	0.0160	0.0090	344	1.778	0.076
	ZT9.5	0.0157	0.0090	344	1.743	0.082
	ZT10.5	0.0180	0.0090	344	2.007	0.046
	ZT11.5	0.0123	0.0090	344	1.369	0.172
	ZT12.5	0.0112	0.0089	344	1.261	0.208
	ZT13.5	0.0186	0.0089	344	2.095	0.037
	ZT14.5	0.0089	0.0090	344	0.992	0.322
	ZT15.5	0.0041	0.0090	344	0.452	0.652
	ZT16.5	0.0048	0.0090	344	0.531	0.596
	ZT17.5	0.0008	0.0090	344	0.090	0.929
	ZT18.5	0.0048	0.0090	344	0.532	0.595
	ZT19.5	0.0020	0.0090	344	0.225	0.822
	ZT20.5	0.0095	0.0091	344	1.043	0.298
	ZT22.5	0.0000	0.0091	344	0.003	0.997
	ZT23.5	0.0021	0.0091	344	0.229	0.819
	Intercept	0.0474	0.0019	366	24.950	0.000
	Night	-0.0083	0.0026	366	-3.187	0.002

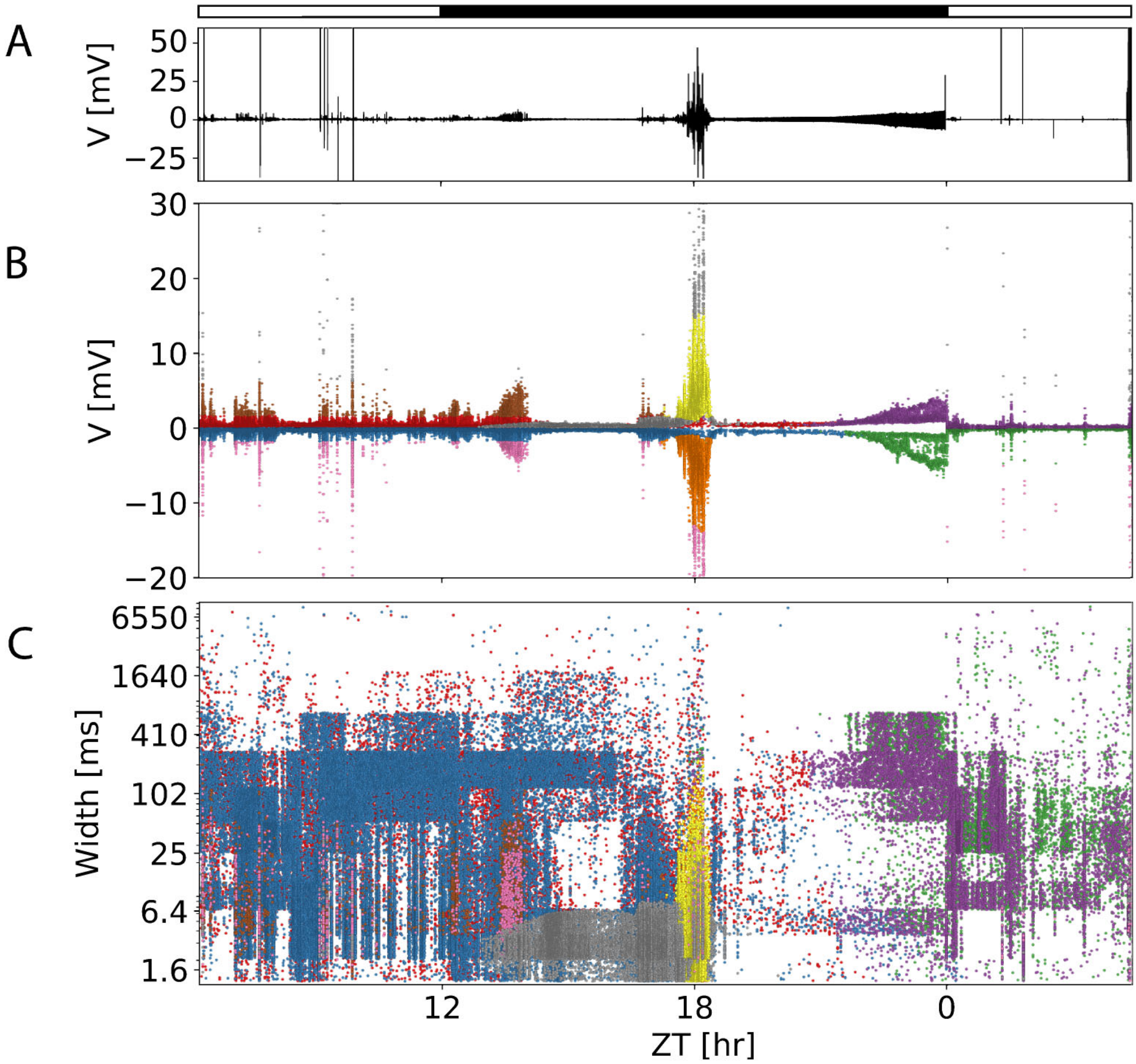

Figure S1 A-C. Event clustering revealed events and episodes that occurred at particular Zeitgeber times. Events can be clustered by similarities to allow for further connectomics analysis to reveal coexisting multiscale dynamics. **(A)** Original loose-patch clamp recording of the cockroach circadian clock circuit *in vivo* over the course of ~ 24 h. The black bar indicates the night from Zeitgeber time (ZT) 12 to ZT 24 (=ZT 0). **(B)** and **(C)** clustering of the events using Bayesian Gaussian mixtures. In contrast to the coarse grain heatmaps, this provides a more sophisticated clustering of events. Clustering was based on amplitude, duration (width), and ZT of the events. ZT was used to account for non-stationarities that may change the properties of the events at much slower rates. Clusters that were separated in time but share characteristics of their events were merged into a single cluster.

Supplementary method information: Comparison with deconvolution methods.

Deconvolution methods have arisen as a mathematically more sophisticated version of template matching for the detection of synaptic events. They rely on extracting or building representative templates from the signal, usually detected using threshold methods (Figure S2, S3, S4). This approach is known to work well when the shapes are consistent across the events [S1], but this is problematic when the shapes are not previously well known or vary due to experimental or biological causes. When deconvolving a certain waveform from a signal containing copies of this waveform embedded in a noisy signal, the resulting signal presents delta-like events in the localization of these copies. This increases the signal-to-noise ratio (SNR) enough to detect the events by applying a threshold, without the sensitivity to Type I and Type II errors.

Signal-to-noise ratio (SNR) is defined as:

$$SNR = \frac{std_{event}}{std_{noise}}$$

Where std_{event} and std_{noise} are the standard deviation of the smallest amplitude event in the synthetic trace and the Gaussian noise, respectively.

A deconvolution based method is shown to work well under a noisy signal which shows originally a SNR = 5 (Figure S3).

In Figures S2 - S7, deconvolution and wavelet transform were qualitative compared in their ability to highlight events presented in synthetic signals. The corresponding signal presented five events of different amplitudes and duration. Here, we presented the cases where SNR = 0.2, SNR = 0.5, SNR = 1, SNR = 2, SNR = 5 and SNR = 10. SNR was based on the amplitude of the smallest event.

Deconvolution: Four deconvolution signals were shown, where each of them using the original waveform without noise that was used to generate each of the events. This was the most favourable situation, the knowledge about the event was perfect, and the resulting deconvolution signals showed a delta-like peak in the corresponding localization of the event. When the duration of the event differed, deconvolved signal showed multiple spurious peaks.

Wavelet transform: For each of the transformed signal, a real Morse wavelet of beta = 2 and gamma = 3 was used. Timescales of the wavelets were selected to be ‘similar’ to events supposed to be detected, but their waveforms were not related to the ones from the events. This compared more to a realistic situation, when the knowledge about the events was limited.

Deconvolution methods failed when noise was comparable in amplitude, even in the cases where the duration of the events was clearly different. Also, the comparison between the signals obtained with different events was not evident in the deconvolution, as it was in the wavelet transform with different scales, for which we had now a criterion to decide to which scale the event was the best.

[REF S1] Merel, J., Shababo, B., Naka, A., Adesnik, H., & Paninski, L. (2016). Bayesian methods for event analysis of intracellular currents. Journal of neuroscience methods, 269, 21–32.
[REF S2] Pernía-Andrade, A. J., Goswami, S. P., Stickler, Y., Fröbe, U., Schlögl, A., & Jonas, P. A deconvolution-based method with high sensitivity and temporal resolution for detection of spontaneous synaptic currents in vitro and in vivo. Biophysical journal, 103(7), 1429–1439.
[REF S3] Guzman, S. J., Schlögl, A., & Schmidt-Hieber, C. (2014). Stimfit: quantifying electrophysiological data with python. Frontiers in neuroinformatics, 8, 16.

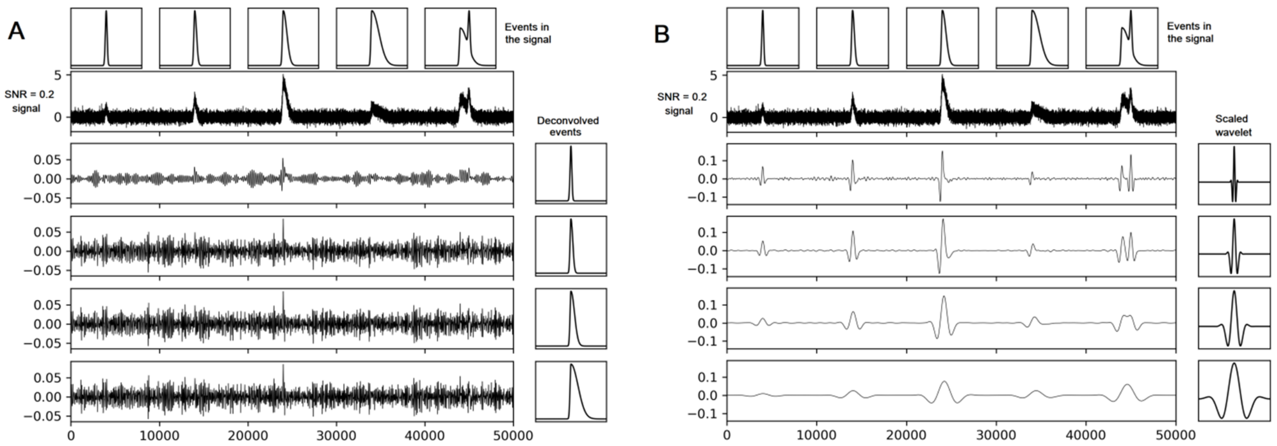

Figure S2. Deconvolution and wavelet transform were qualitatively compared in their ability to highlight events presented in a synthetic signal of SNR = 0.2. **(A):** Four deconvolution signals were shown, each of them using the original waveform without noise that was used to generate each of the events. **(B):** For each of the transformed signal, a real Morse wavelet of beta = 2 and gamma = 3 was used. Timescales of the wavelets were selected to be ‘similar’ to the events supposed to be detected, but their waveforms were not related to the ones from the events. Time and amplitude axes are in arbitrary units.

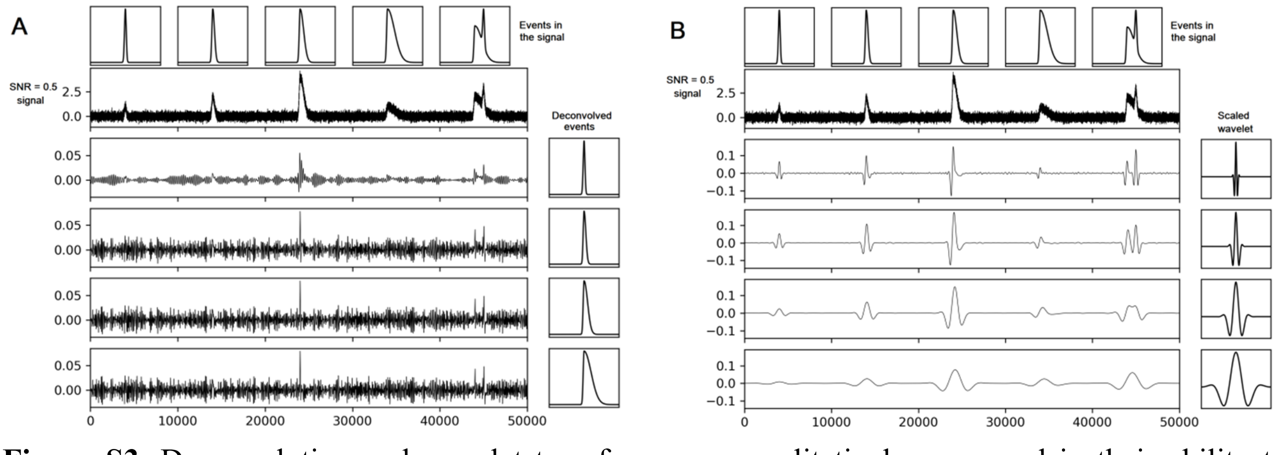

Figure S3. Deconvolution and wavelet transform were qualitatively compared in their ability to highlight events presented in a synthetic signal of SNR = 0.5. **(A):** Four deconvolution signals were shown, each of them using the original waveform without noise that was used to generate each of the events. **(B):** For each of the transformed signal, a real Morse wavelet of beta = 2 and gamma = 3 was used. Timescales of the wavelets were selected to be ‘similar’ to the events supposed to be detected, but their waveforms were not related to the ones from the events. Time and amplitude axes are in arbitrary units.

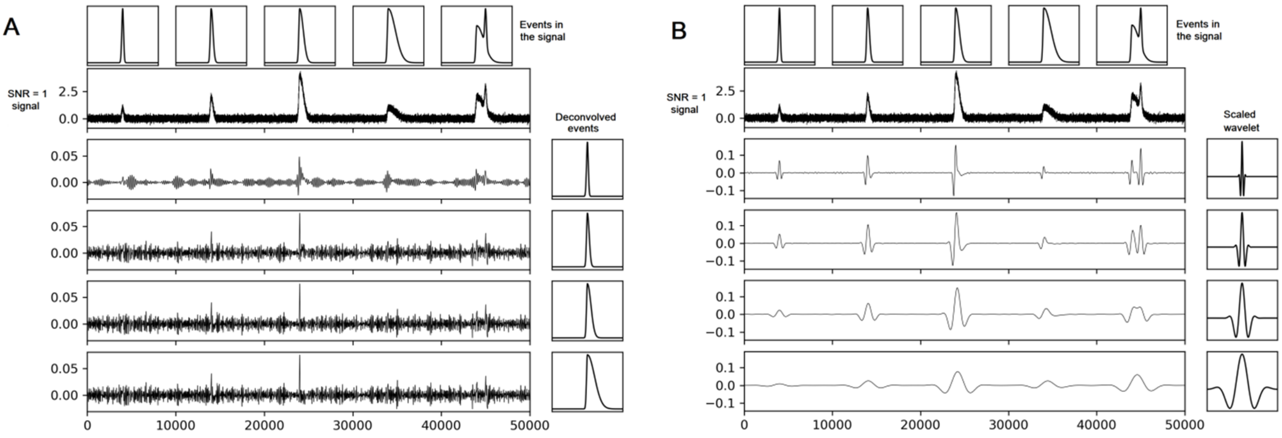

Figure S4. Deconvolution and wavelet transform were qualitatively compared in their ability to highlight events presented in a synthetic signal of SNR = 1. **(A):** Four deconvolution signals were shown, each of them using the original waveform without noise that was used to generate each of the events. **(B):** For each of the transformed signal, a real Morse wavelet of beta = 2 and gamma = 3 was used. Timescales of the wavelets were selected to be 'similar' to the events supposed to be detected, but their waveforms were not related to the ones from the events. Time and amplitude axes are in arbitrary units.

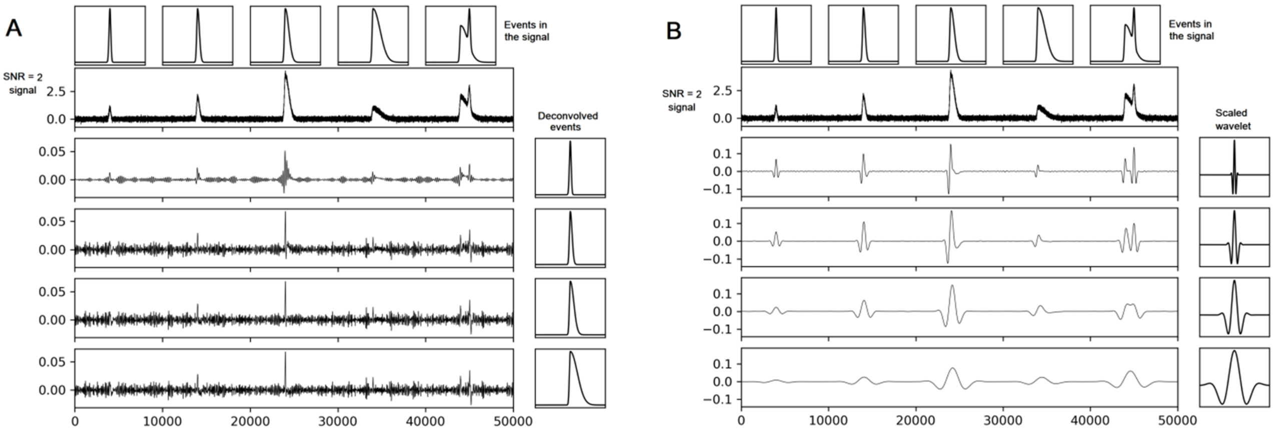

Figure S5. Deconvolution and wavelet transform were qualitatively compared in their ability to highlight events presented in a synthetic signal of SNR = 1. **(A):** Four deconvolution signals were shown, each of them using the original waveform without noise that was used to generate each of the events. **(B):** For each of the transformed signal, a real Morse wavelet of beta = 2 and gamma = 3 was used. Timescales of the wavelets were selected to be 'similar' to the events supposed to be detected, but their waveforms were not related to the ones from the events. Time and amplitude axes are in arbitrary units.

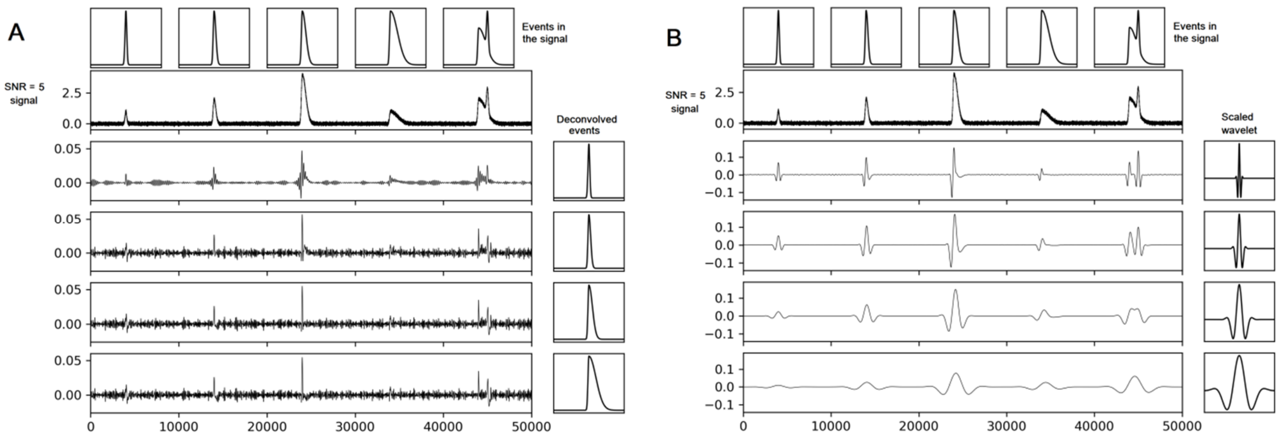

Figure S8. Deconvolution and wavelet transform were qualitatively compared in their ability to highlight events presented in a synthetic signal of SNR = 5. **(A):** Four deconvolution signals were shown, each of them using the original waveform without noise that was used to generate each of the events. **(B):** For each of the transformed signal, a real Morse wavelet of beta = 2 and gamma = 3 was used. Timescales of the wavelets were selected to be 'similar' to the events supposed to be detected, but their waveforms were not related to the ones from the events. Time and amplitude axes are in arbitrary units.

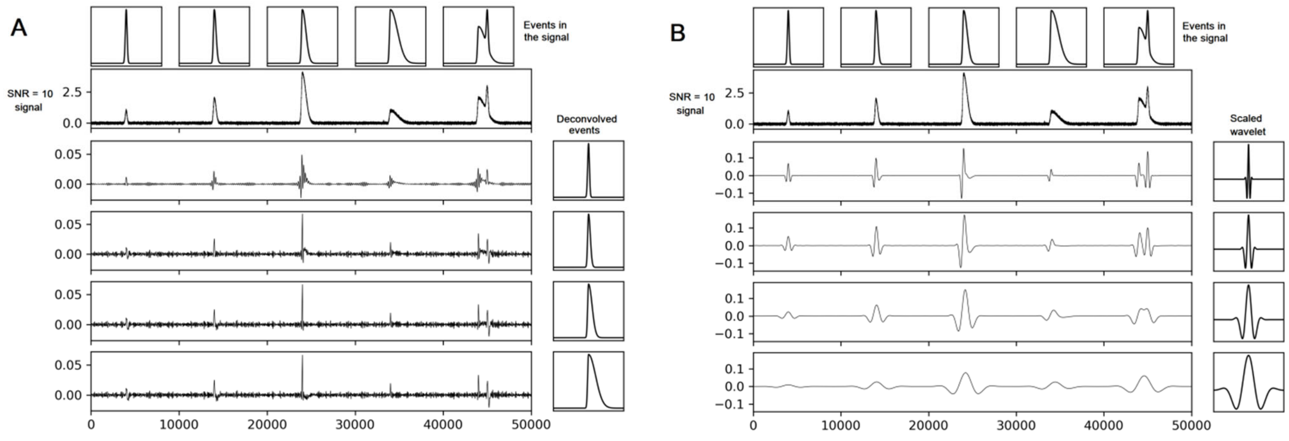

Figure S7. Deconvolution and wavelet transform were qualitatively compared in their ability to highlight events presented in a synthetic signal of SNR = 10. **(A):** Four deconvolution signals were shown, each of them using the original waveform without noise that was used to generate each of the events. **(B):** For each of the transformed signal, a real Morse wavelet of beta = 2 and gamma = 3 was used. Timescales of the wavelets were selected to be 'similar' to the events supposed to be detected, but their waveforms were not related to the ones from the events. Time and amplitude axes are in arbitrary units.
